# Quantitative multiplexed proteomics analysis reveals reshaping of the lysine 2-hydroxyisobutyrylome in *Fusarium graminearum* by tebuconazole

**DOI:** 10.1186/s12864-022-08372-4

**Published:** 2022-02-18

**Authors:** Yanxiang Zhao, Limin Zhang, Chao Ju, Xiaoyan Zhang, Jinguang Huang

**Affiliations:** 1 College of Plant Health and Medicine and Key Lab of Integrated Crop Disease and Pest Management of Shandong Province, Qingdao Agricultural University, Qingdao, 266109 Shandong Province China; 2 College of Agriculture, Ludong University, Yantai, 264025 Shandong Province China

**Keywords:** Posttranslational modification, 2-hydroxyisobutyrylome, *Fusarium* head blight, *Fusarium graminearum*, Fungicide resistance

## Abstract

**Backgrounds:**

Lysine 2-hydroxyisobutyrylation (Khib) is a newly discovered posttranslational modification (PTM) and has been identified in several prokaryotic and eukaryotic organisms. *Fusarium graminearum*, a major pathogen of *Fusarium* head blight (FHB) in cereal crops, can cause considerable yield loss and produce various mycotoxins that threaten human health. The application of chemical fungicides such as tebuconazole (TEC) remains the major method to control this pathogen. However, the distribution of Khib in *F. graminearum* and whether Khib is remodified in response to fungicide stress remain unknown.

**Results:**

Here, we carried out a proteome-wide analysis of Khib in *F. graminearum*, identifying the reshaping of the lysine 2-hydroxyisobutyrylome by tebuconazole, using the most recently developed high-resolution LC–MS/MS technique in combination with high-specific affinity enrichment. Specifically, 3501 Khib sites on 1049 proteins were identified, and 1083 Khib sites on 556 modified proteins normalized to the total protein content were changed significantly after TEC treatment. Bioinformatics analysis showed that Khib proteins are involved in a wide range of biological processes and may be involved in virulence and deoxynivalenol (DON) production, as well as sterol biosynthesis, in *F. graminearum*.

**Conclusions:**

Here, we provided a wealth of resources for further study of the roles of Khib in the fungicide resistance of *F. graminearum*. The results enhanced our understanding of this PTM in filamentous ascomycete fungi and provided insight into the remodification of Khib sites during azole fungicide challenge in *F. graminearum*.

**Supplementary Information:**

The online version contains supplementary material available at 10.1186/s12864-022-08372-4.

## Introduction

Protein posttranslational modifications (PTMs), such as methylation, acetylation, and phosphorylation, play important roles in various biological events and cellular processes. Extensive studies in recent decades have revealed more than 200 different types of PTMs. Due to their unstable basic side chain, lysine residues are more prone to modification than other residues, and lysine acylation is one of the most common PTMs. Lysine 2-hydroxyisobutyrylation (Khib), a unique acylation, was first reported as a new type of histone marker in eukaryotic cells [1]. Recent studies have shown that this modification is evolutionarily conserved across bacteria, yeast, plants, animals, and humans on both histone and nonhistone proteins [1–7]. Recently, the 2-hydroxyisobutyrylomes of three filamentous fungi, namely, *Ustilaginoidea virens*, *Botrytis cinerea,* and *Fusarium oxysporum*, were also reported [8–10]. Khib adds a large 2-hydroxyisobutyryl group to the lysine residue, which increases the size of the lysine side chain, abolishes the positive charge, and introduces a hydroxyl group that enables the modified lysine to form hydrogen bonds with other molecules [1]. As a result, Khib can influence various biological processes, such as the regulation of gene expression, energy metabolism, protein translation, and secondary metabolism [1, 11–13].

*Fusarium graminearum* is the major causal agent of *Fusarium* head blight (FHB), a devastating disease of wheat and barley worldwide. In addition, *F. graminearum* can produce various toxins, including zearalenone (ZEA) and trichothecenes, such as deoxynivalenol (DON), nivalenol (NIV) and their acetylated derivatives [14]. Contamination of grains with mycotoxins produced by *F. graminearum* poses a threat to the health of humans and animals and leads to challenges in grain storage. Given its great impact on global grain production and food safety, *F. graminearum* was designated one of the top ten plant fungal pathogens by the international research community [15, 16]. In agricultural practice, the application of chemical agents such as tebuconazole (TEC) and carbendazim (BCM) remains the major method to control this disease. Unfortunately, fungicide resistance is emerging in *F. graminearum* due to the abuse and long-term application of these substances. Extensive research on the mechanism of fungicide resistance in *F. graminearum* has been performed, but only a few studies on how PTMs participate in fungicide resistance have been reported.

The application of “omics” approaches, including transcriptomics, has provided insights for deciphering how *F. graminearum* copes with fungicide stress [17–19]. Comparative transcriptomics and experimental evidence in other fungal studies revealed that transporter genes were involved in different levels of sensitivity to fungicide treatment [20, 21]. Proteomics has also been frequently employed in research on *F. graminearum*, especially in the discovery of virulence factors and in gene function studies [22–25]. However, there are only a few reports about the influence of fungicide treatment on the proteome of *F. graminearum* [19, 26]. To gain further insights into the fungal response to fungicide, we employed cutting-edge proteomics technologies, combining high-specific affinity enrichment and high-resolution mass spectrometry (MS), to identify the proteins harboring Khib sites in *F. graminearum.* Multiplexed proteomics using different isobaric tags was applied to quantify changes in the lysine 2-hydroxyisobutyrylome after treatment with TEC. This study provides a comprehensive profile of the lysine 2-hydroxyisobutyrylome of *F. graminearum* and provides new insights into fungicide resistance in *F. graminearum* and disease control of FHB.

## Results and discussion

### Alteration of Khib in *Fusarium graminearum* by fungicides

**Fig. 1:**
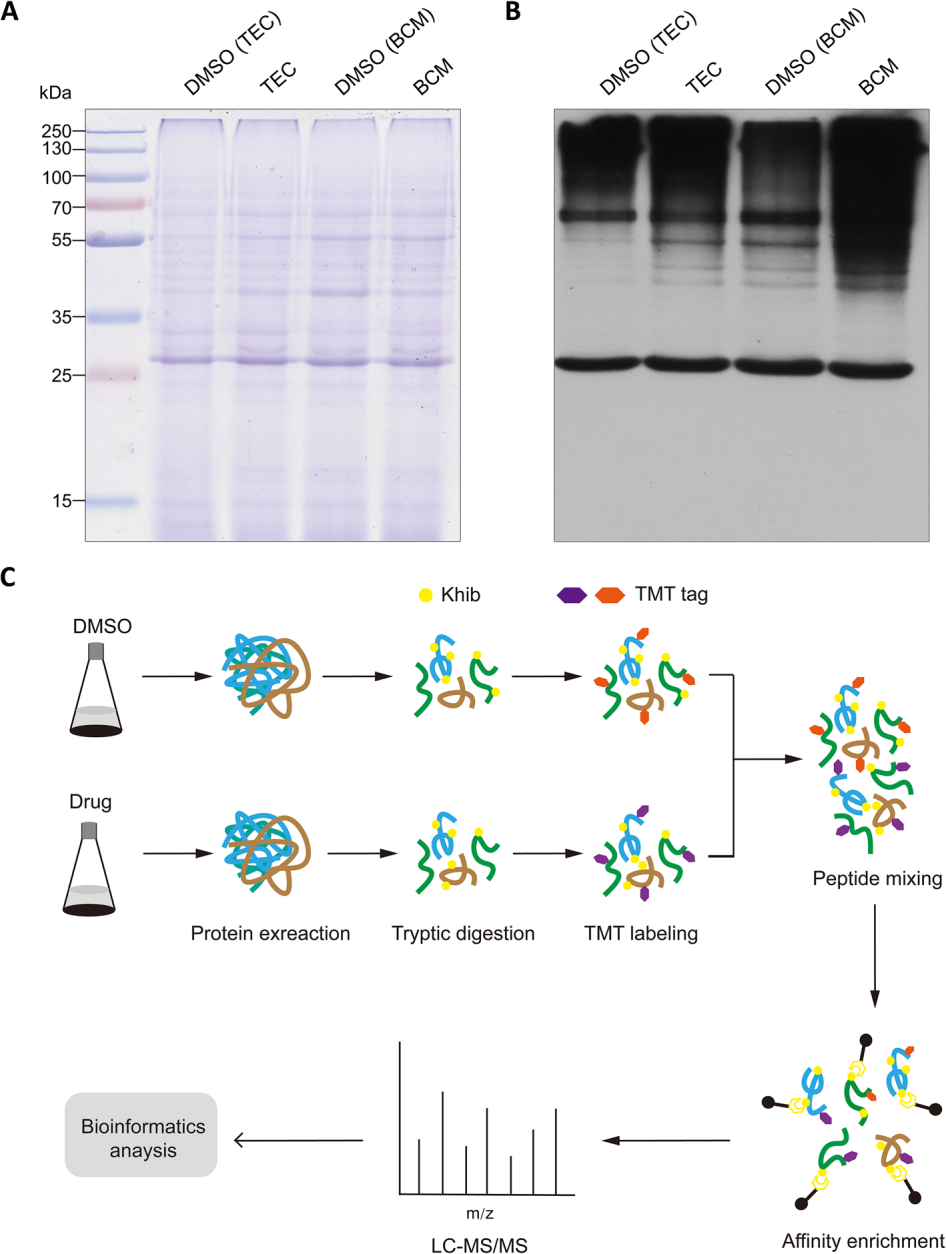
Detection of lysine 2-hydroxyisobutyrylation in *F. graminearum.* **A** SDS-PAGE gel stained with Coomassie blue was used as a loading control. Total proteins in *F. graminearum* treated with tebuconazole (TEC), carbendazim (BCM) or an equal volume of DMSO as control were loaded in each lane. **B** Western blotting analysis of the samples in (**A**) was performed with pan-2-hydroxyisobutyryllysine antibody. The bands corresponding to the samples in (**A**) were shown. **C** Schematic illustration of the proteomic analytical steps for mycelium collection, protein extraction, tryptic digestion, TMT labeling, affinity enrichment, LC–MS/MS and bioinformatics analysis

To investigate whether the status of Khib was altered after fungicide treatment, we treated *F. graminearum* with two fungicides, namely, TEC and BCM, which are commonly used fungicides in FHB control. Western blotting of the total protein extracts of *F. graminearum* with or without fungicide treatment with a pan anti-2-hydroxyisobutyryllysine antibody was performed. The results showed that multiple protein bands larger than 25 kDa were detected in all the samples, indicating that Khib is widely distributed in *F. graminearum.* Interestingly, obvious enhancement of the blotted proteins in samples treated with the fungicide compared to control samples with equal amounts of DMSO was observed in each fungicide trial (Fig. 1A). This demonstrates that the activity of Khib is enhanced in response to fungicide treatment in *F. graminearum*. This also suggests that fungicides may result in changes in the lysine 2-hydroxyisobutyrylome.

### Identification of Khib peptides involved in the fungicide response in *F. graminearum*

**Fig. 2:**
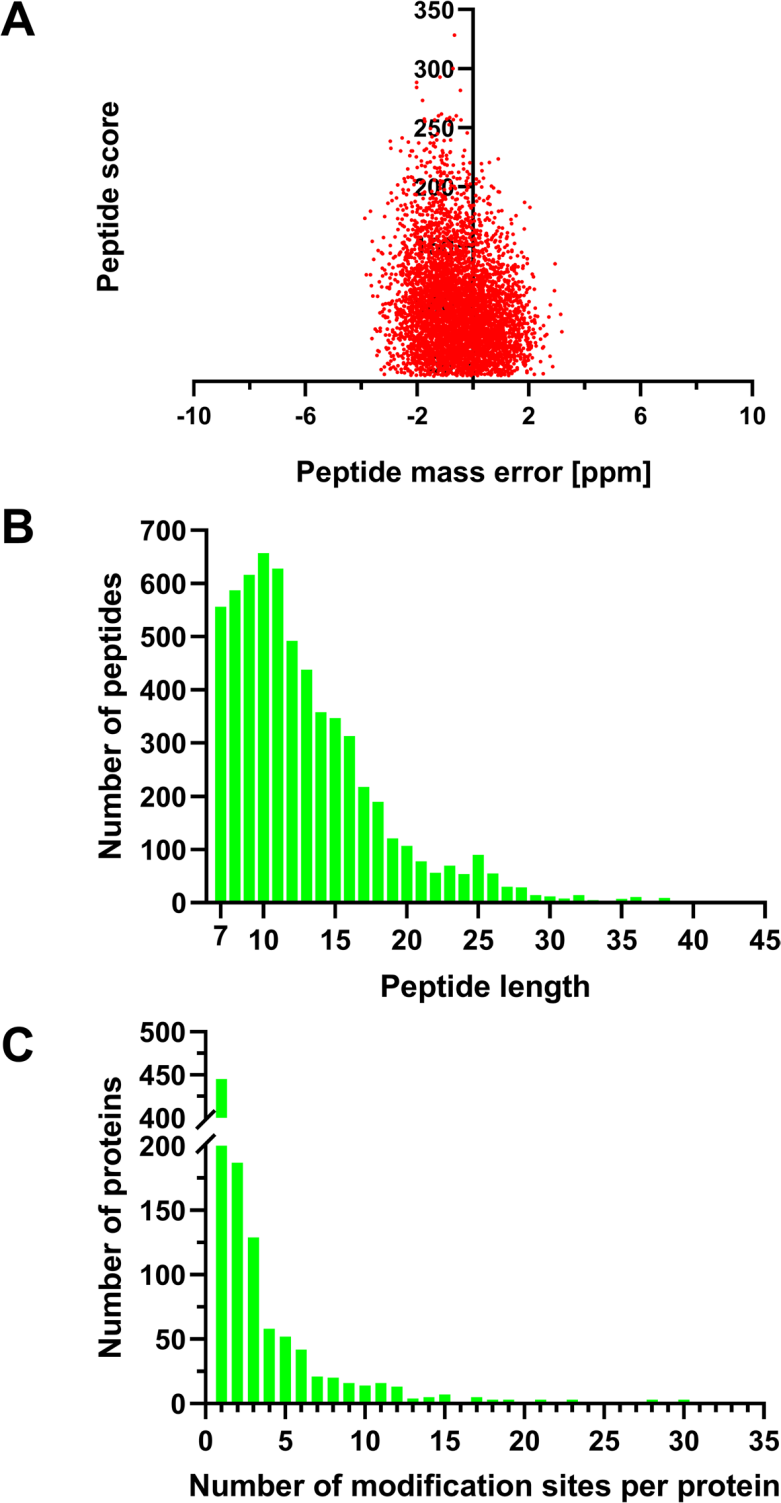
Quality control validation of MS data. **A** Mass error distribution of all identified peptides. **B** Length distribution of all identified peptides. **C** Distribution of the number of Khib modification sites per protein. Detailed data are listed in Additional File 1: Table 1

Quantitative multiplexed proteomics using TMT labeling was applied for global profiling of the lysine 2-hydroxyisobutyrylome in response to fungicide treatment (Fig. 1B). To check the quality of the MS data, the mass errors of all identified peptides were analyzed. The mass errors of most of the identified peptides were less than 3 ppm, suggesting that the MS data were of high quality (Fig. 2A; Additional File 1: Table S1). The length of most peptides (91%) was between 7 and 20 amino acids (Fig. 2B), which was consistent with the length distribution of peptides digested by trypsin. In total, we identified 3807 unique peptides, of which 3492 were Khib modified. Finally, 3501 Khib sites on 1049 proteins were identified, where 3035 modification sites on 937 proteins were quantifiable (Additional File 1: Table S1). The proportion of identified Khib proteins in *F. graminearum* is 7.4% (1049/14160), similar to another *Fusarium* species, *F. oxysporum,* in which 3782 Khib sites on 1299 proteins have been identified, accounting for 7.3% of the total proteins in *F. oxysporum*. The identified proteins contained various numbers of Khib sites. Only approximately 42.4% of the proteins (445 proteins) had one Khib site, and 87% of the proteins (913 proteins) contained fewer than 6 Khib sites (Fig. 2C). FGRAMPH1_01T23287 and FGSG_07375 have as many as 30 Khib sites in the identified proteins. Compared with the lysine acetylome in *F. graminearum* reported by Zhou et al. [27], the identified Khib proteins in this study were much more abundant than the reported acetylated proteins (1049 vs. 364), and the average number of Khib sites per protein was also larger than that of acetylated sites per protein (3.3 vs. 1.6). This result indicated that Khib is much more abundant than lysine acetylation (Kac) in *F. graminearum*.

### Motif analysis of 2-hydroxyisobutyrylated lysine in *F. graminearum*

**Fig. 3:**
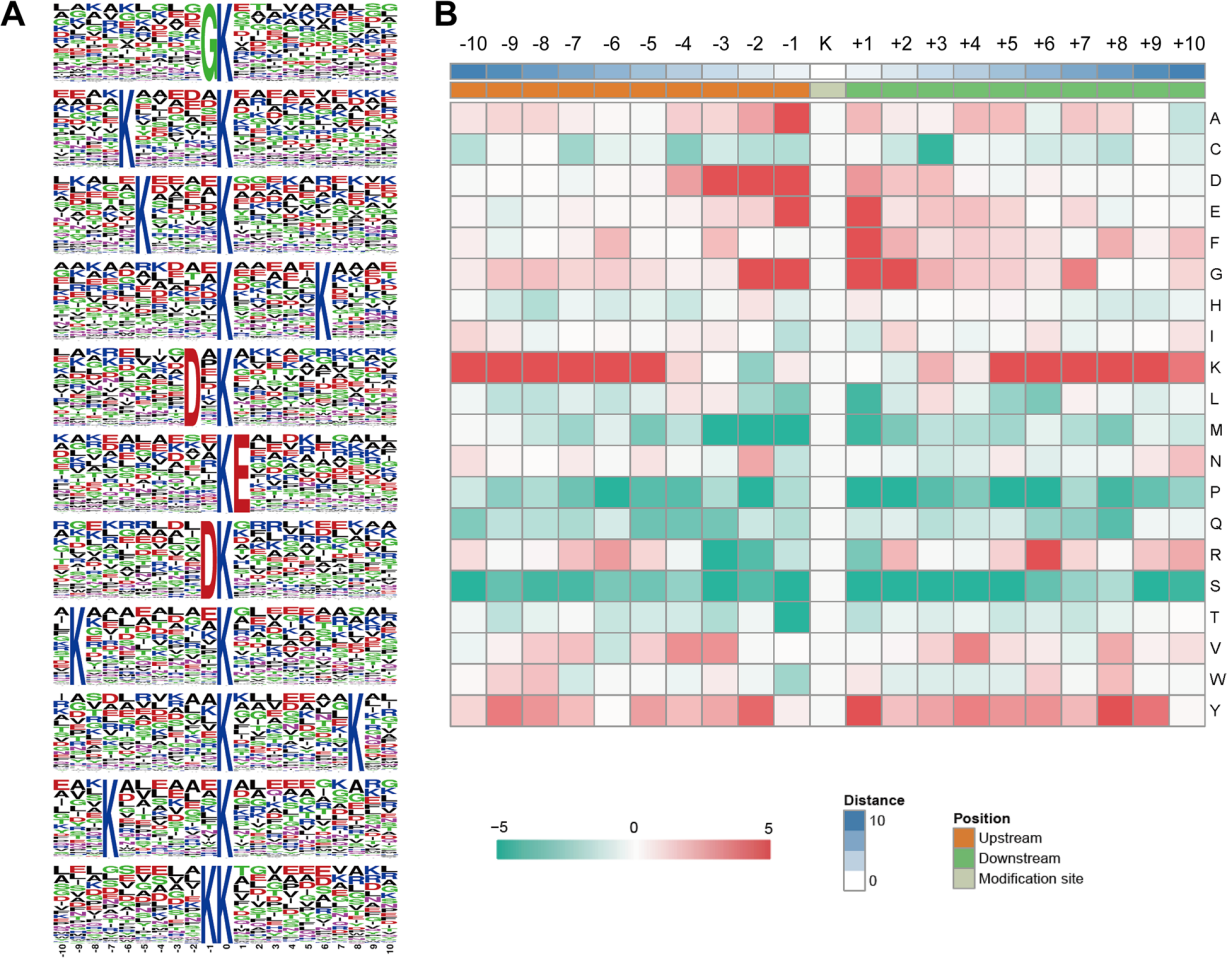
Motif analyses of the Khib peptides. **A** Sequence logo of Khib motifs and conservation of Khib sites identified by the Motif-X program. The motif shows the Khib sites at the center and 10 amino acids upstream and downstream of the Khib site. The motifs with the significance of *p* < 0.000001 are shown. **B** Heatmap of the amino acid compositions of Khib sites. The intensity map shows the relative abundance of ± 10 amino acids from the Khib site. Colors represent the log10 of the ratio of frequencies within Khib-21-mers versus non-Khib-21-mers (red shows enrichment, green shows depletion)

To clarify the amino acid residue preference around the Khib sites, we analyzed the motif patterns in the identified proteins using the Motif-X program. As shown in Fig. 3, the nearest position adjacent to the Khib site is less well conserved, which is different from the acetylation motif reported in *F. graminearum* but is similar to the Khib motif pattern reported in other organisms [3–7]. Among the 11 significantly enriched motifs, only five motifs with amino acids at positions -1, -2 or + 1 were overrepresented, namely, the GK, DxK, DK, KK, and KE motifs. Peptides with these motifs accounted for 46.7% of all the identified peptides. The GK motif had the highest proportion, accounting for 16.1%. All these motifs except GK are also present in developing rice seeds [3]. According to the heatmap, the frequencies of alanine (A), aspartic acid (D), glutamic acid (E), and glycine (G) were the highest at position -1, while glutamic acid (E), phenylalanine (F), glycine (G) and tyrosine (Y) were most abundant at position + 1. The amino acid preference at position -1 in *F. graminearum* is similar to that in *Physcomitrella patens* but is different from that in *Toxoplasma gondii*, while the preference at position + 1 varies [4, 6]. Lysine residues were overrepresented at positions -10 to -5 and + 5 to + 10, which has been observed in most reported 2-hydroxyisobutyrylatomes. The specific Khib motif patterns indicate unique substrate preferences in *F. graminearum*.

### Conservation analysis of Khib sites

**Fig. 4:**
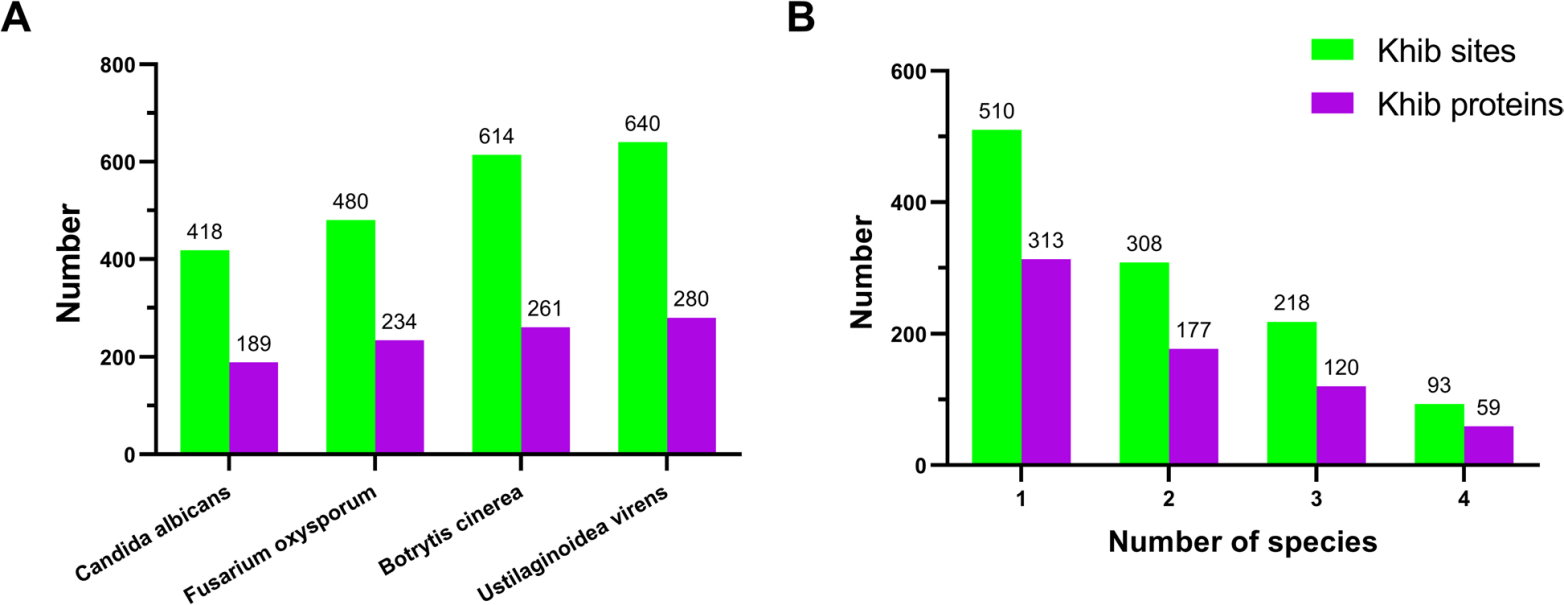
Conservation analysis of Khib sites. **A** The number of identified Khib sites of *F. graminearum* which are conserved in *Candida albicans*, *Fusarium oxysporum*, *Botrytis cinerea*, and *Ustilaginoidea virens,* and the number of orthologous proteins with these sites. The orthologs were identified by BLASTP using Khib protein of *F. graminearum* as queries, and the equivalent sites were then identified by sequence alignment using MUSCLE. **B** The analysis of Khib sites and proteins with these sites conserved in the above five fungi. The x-axis indicates the number of species that possess equivalent Khib sites of an *F. graminearum* Khib site. The y-axis shows the number of Khib sites in *F. graminearum*. Detailed data are listed in Additional File 2: Table S2

To date, the 2-hydroxyisobutyrylomes of several fungi have been reported, including *Ustilaginoidea virens*, *Botrytis cinerea, Fusarium oxysporum, Candida albicans* [8–10, 12]*.* To understand the evolutionary conservation of lysine 2-hydroxyisobutyrylation in different species, identified Khib proteins and sites of *F. graminearum* were compared with those from the above four fungi by using BLASTp to identify orthologs followed by using MUSCLE to make alignments. In total, 1129 Khib sites on 430 Khib proteins in *F. graminearum* were identified with equivalent sites in at least one 2-hydroxyisobutyrylatome of the other four fungi, accounting for 32.3% of the total Khib sites and 41% of Khib proteins identified in *F. graminearum* (Fig. 4; Additional File 2: Tables S2). The number of orthologs with equivalent Khib sites in *U. virens*, *B. cinerea, F. oxysporum, and C. albicans* were 280, 261, 234, and 189, respectively (Fig. 4A). The proportions of proteins with orthologs in the three plant pathogenic fungi (*U. virens*, *B. cinerea,* and *F. oxysporum*) were 22.3%—26.7%, and in yeast *C. albicans* was 18%, which is slightly lower than the former. There were 168 sites on 92 proteins conserved in the four phytopathogenic fungi, while only 93 Khib sites on 59 proteins were well conserved in all five fungi. As shown in Fig. 4B, most Khib sites identified in this analysis have equivalent sites in only one other fungus, which accounts for 45.2% of the total identified Khib sites (510/1129).

### Characterization of Khib proteins in *F. graminearum*

**Fig. 5:**
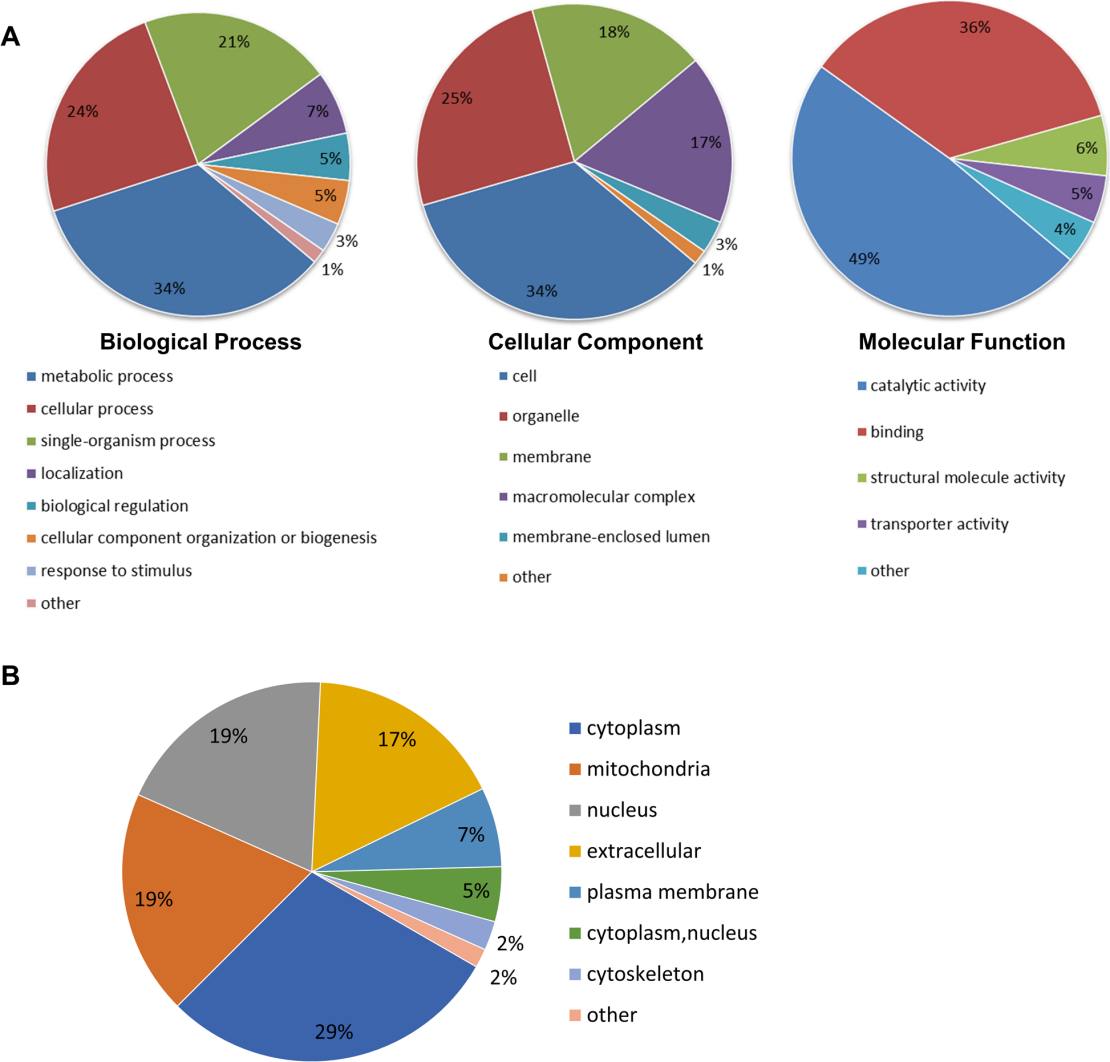
Functional annotation and subcellular localization of all the identified Khib proteins in *F. graminearum*. **A** Pie charts showing GO annotations of all the identified Khib proteins based on biological process (BP), cellular component (CC), and molecular function (MF). The percentage of each GO term represents the proportion of proteins in the respective group. **B** Subcellular localization analysis of Khib proteins predicted by WoLF PSORT software in *F. graminearum*

To comprehensively elucidate the possible function and localization of Khib proteins in *F. graminearum*, we performed GO classification with all the identified Khib proteins based on biological processes, molecular functions, and cellular components. Most of the Khib proteins were classified into three biological process groups, namely, metabolic process (34%), cellular process (24%) and single-organism process (21%), and were associated with catalytic activity (49%) and binding (36%) in terms of molecular function. In terms of cellular components, the Khib proteins were mainly distributed in cells (34%), organelles (25%), membranes (18%) and macromolecular complexes (17%) (Fig. 5A). The subcellular localization of the Khib proteins was also predicted with the Wolf PSORT program. The Khib proteins were distributed in the cytoplasm (29.17%), mitochondria (19.16%), nucleus (19.07%) and extracellular space (17.06%) (Fig. 5B). The GO classification of the Khib proteins in *F. graminearum* was similar to that in other reported organisms and was also similar to that of the acetylated proteins reported in *F. graminearum*. Compared to the acetylome of *F. graminearum*, the subcellular localization analysis showed that a greater proportion of modified proteins was localized in the extracellular space in the 2-hydroxyisobutyrylome.

### Functional enrichment analysis of Khib proteins in *F. graminearum*

**Fig. 6:**
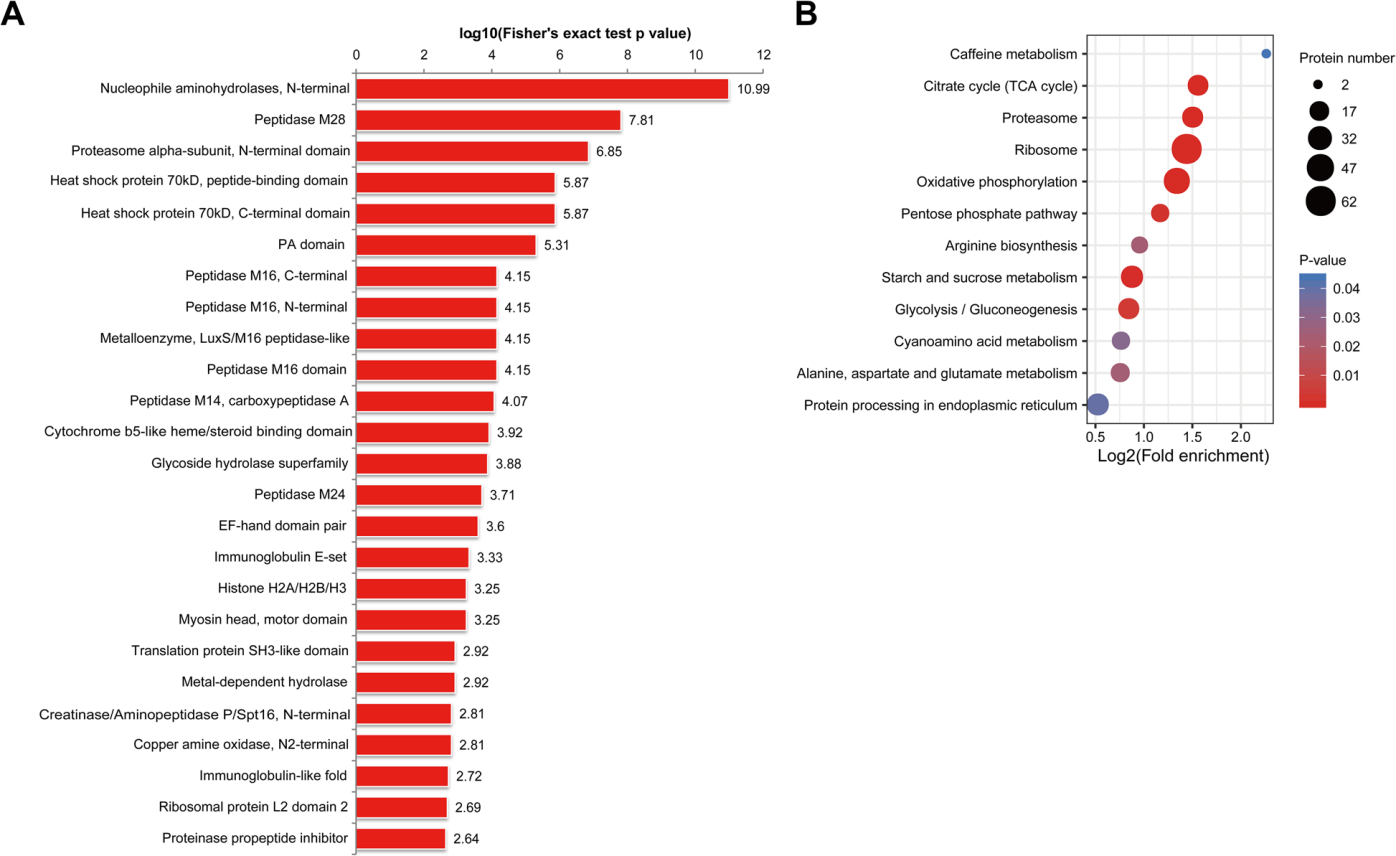
Enrichment analysis of all the identified Khib proteins in *F. graminearum*. **A** Protein domain enrichment analysis. The InterPro domain database was used in the analysis. The -log10(Fisher’s exact *p*-value) is shown on the x-axis. Protein domains with a *p*-value < 0.05 were considered significant. Detailed data are listed in Additional File 4: Table S4. **B** KEGG pathway enrichment analysis (*p*-value < 0.05)*.* The size of the circle represents the protein number, and the color gradient (from red to light blue) represents the corrected p-value (0–0.05). Detailed data are listed in Additional File 5: Table S5

To better understand Khib in *F. graminearum*, GO, KEGG, and protein domain enrichment analyses of the Khib proteins were carried out (Fig. 6, Additional File 3–5: Tables S3, S4 and S5). GO-based enrichment analysis showed that the modified proteins were mainly significantly enriched in 14 biological process terms, such as peptide metabolic process, translation, peptide biosynthetic process and amide biosynthetic process, and enriched in 8 cellular component terms, including the cytoplasm, cell, intracellular, and ribosome parts. In terms of molecular function, structural constituents of ribosomes and various enzymatic activities (such as exopeptidase activity, peptidase activity, and hydrolase activity) were significantly enriched (Additional File 10: Figure S1 and Additional File 3: Table S3). In the protein domain enrichment analysis, 25 protein domains were identified, and these domains were also related to diverse enzymes, such as peptidase, hydrolase, and oxidase (Fig. 6A, Additional File 4: Table S4). All the results suggested that proteins with Khib modification were involved in various cellular and metabolic processes. The KEGG pathway enrichment analysis also suggested this. A large number of Khib proteins were enriched in 11 significant pathways, including the ribosome (fgr03010), oxidative phosphorylation (fgr00190), citrate cycle (TCA cycle, fgr00020), starch and sucrose metabolism (fgr00500), and proteasome (fgr03050) pathways (Fig. 6B, Additional File 5: Table S5). Furthermore, a protein–protein interaction network with 583 nodes and 4800 interactions was constructed, and cluster analysis identified the 5 most abundant clusters, namely, the ribosome subnetwork, oxidative phosphorylation subnetwork, proteasome subnetwork, spliceosome subnetwork, and aminoacyl-tRNA biosynthesis subnetwork (Additional File 10: Figure S2). These enrichment analysis results were similar to previous reports in other organisms, which implies that Khib may have some conserved roles in different organisms.

### Reshaping of the lysine 2-hydroxyisobutyrylome of *F. graminearum *by TEC treatment

**Fig. 7:**
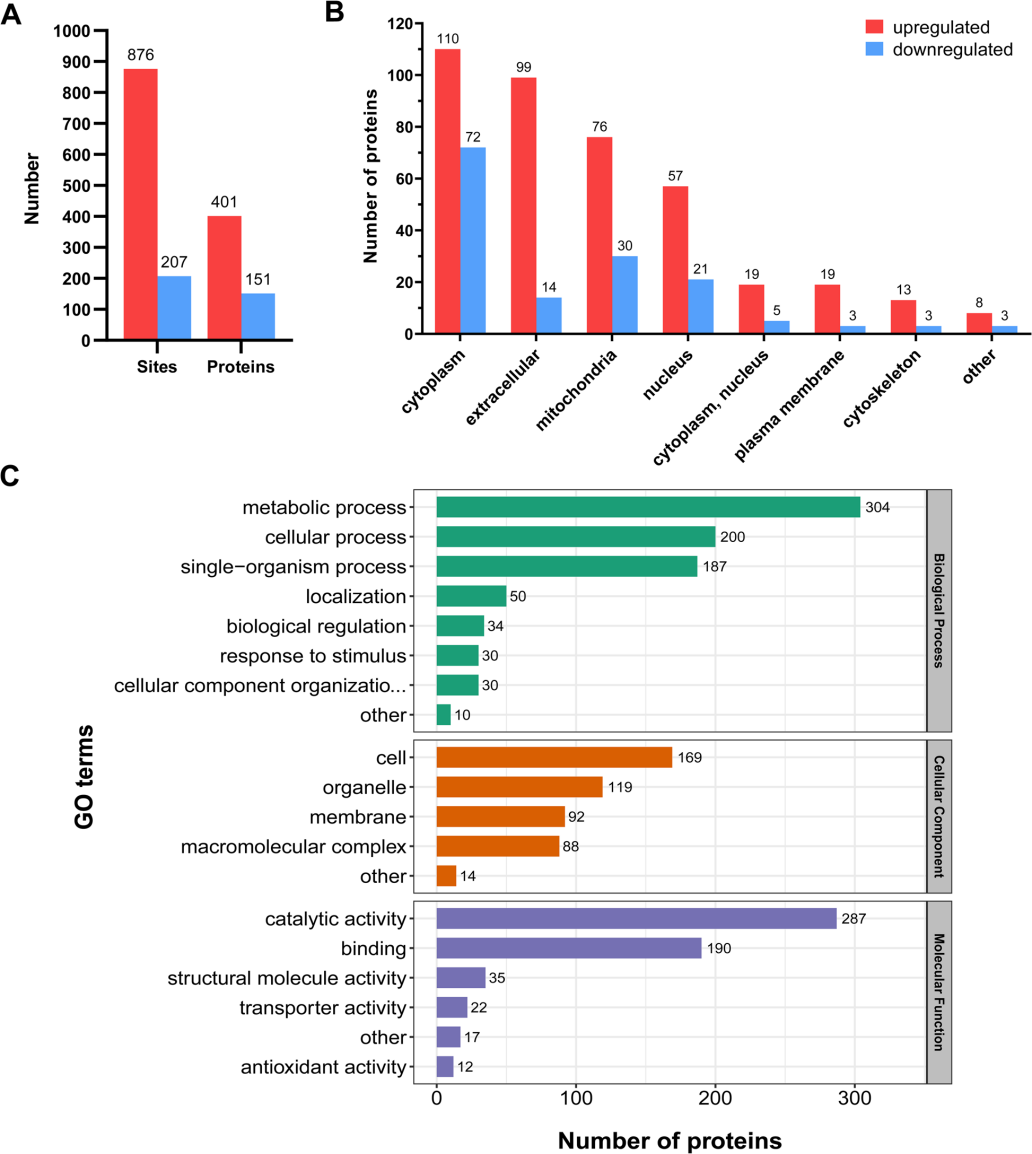
Characterization of tebuconazole-affected Khib proteins (TAKPs) in *F. graminearum*. **A** The number of identified Khib sites and Khib proteins affected by tebuconazole. **B** The subcellular localization of TAKPs predicted by WoLF PSORT. The number of TAKPs in each compartment is shown above the bar. (C) GO classification of TAKPs. The number of TAKPs at GO term level 2 is shown beside the bar. Detailed data are listed in Additional File 6: Table S6

TEC-treated and control samples (TEC vs. TEC_CK) were labeled with different TMT tags prior to LC–MS/MS. After the fragmentation of TMT tags in MS2, the complement reporter ions were detected and quantified to determine the change in Khib peptides between TEC and TEC_CK samples. With the criterion of fold change greater than 1.3, 1083 Khib sites of 509 proteins, normalized to total proteins, were considered significantly affected by TEC treatment. Specifically, Khib on 876 sites of 401 proteins was enhanced, while Khib on 207 sites of 151 proteins was suppressed. In addition, 43 proteins showed both upregulation and downregulation of Khib (Fig. 7A, Additional File 6: Table S6). The results indicated that the overall Khib in *F. graminearum* was enhanced after TEC treatment, which was consistent with the Western blotting results (Fig. 1). The ten sites accounting for the highest and lowest changes in Khib proportion are listed in Table 1.

**Table 1:** Top ten sites with the highest and lowest ratios (TEC/TEC_CK) in the estimation of the change in Khib in response to tebuconazole

Locus	Khib Position	TEC/TEC_CK Ratio	Regulation Type	Protein Description/Predicted Function
FGSG_07822	290	4.23	Up	Serine protease
FGRAMPH1_01T19927	123	4.06	Up	Glucoamylase
FGSG_00639	401	3.94	Up	Tubulin alpha chain, TUB1
FGSG_04022	177	3.67	Up	Putative amidase
FGSG_04289	80	3.67	Up	Histone H4
FGSG_08723	259	3.33	Up	Transaldolase
FGSG_02328	641	3.29	Up	Multicopper oxidase GIP1
FGSG_05906	265	3.16	Up	Lipase
FGRAMPH1_01T05035	331	3.14	Up	Clavaminate synthase-like protein
FGSG_01574	326	3.12	Up	Autophagy-related protein 27, ATG27
FGSG_09532	317	0.42	Down	3'(2'),5'-bisphosphate nucleotidase
FGSG_04410	201	0.41	Down	Proteasome subunit alpha type
FGRAMPH1_01T24163	303	0.41	Down	Protein disulfide-isomerase erp38
FGRAMPH1_01T06765	195	0.38	Down	5'-amp-activated protein kinase subunit beta-2
FGSG_05177	90	0.38	Down	Perilipin mpl1
FGSG_06035	120	0.36	Down	Cell division control protein
FGSG_02770	114	0.34	Down	Fructose-bisphosphate aldolase
FGSG_01099	132	0.32	Down	GTP-binding nuclear protein
FGSG_02783	60	0.31	Down	Sterol 24-C-methyltransferase
FGSG_02770	308	0.31	Down	Fructose-bisphosphate aldolase

To further understand the distribution of proteins with identified changes in Khib caused by TEC, we performed a subcellular localization analysis. Similar to the total Khib protein distribution, the TEC-affected Khib proteins (TAKPs) were also mainly located in the cytoplasm (32.2%), extracellular space (20.8%), mitochondria (18.7%), and nucleus (14.7%). In all the compartments, the upregulated Khib proteins were always more abundant than the suppressed Khib proteins. Remarkably, in the extracellular space, the upregulated Khib proteins were disproportionately more abundant than the downregulated Khib proteins (Fig. 7B).

**Fig. 8:**
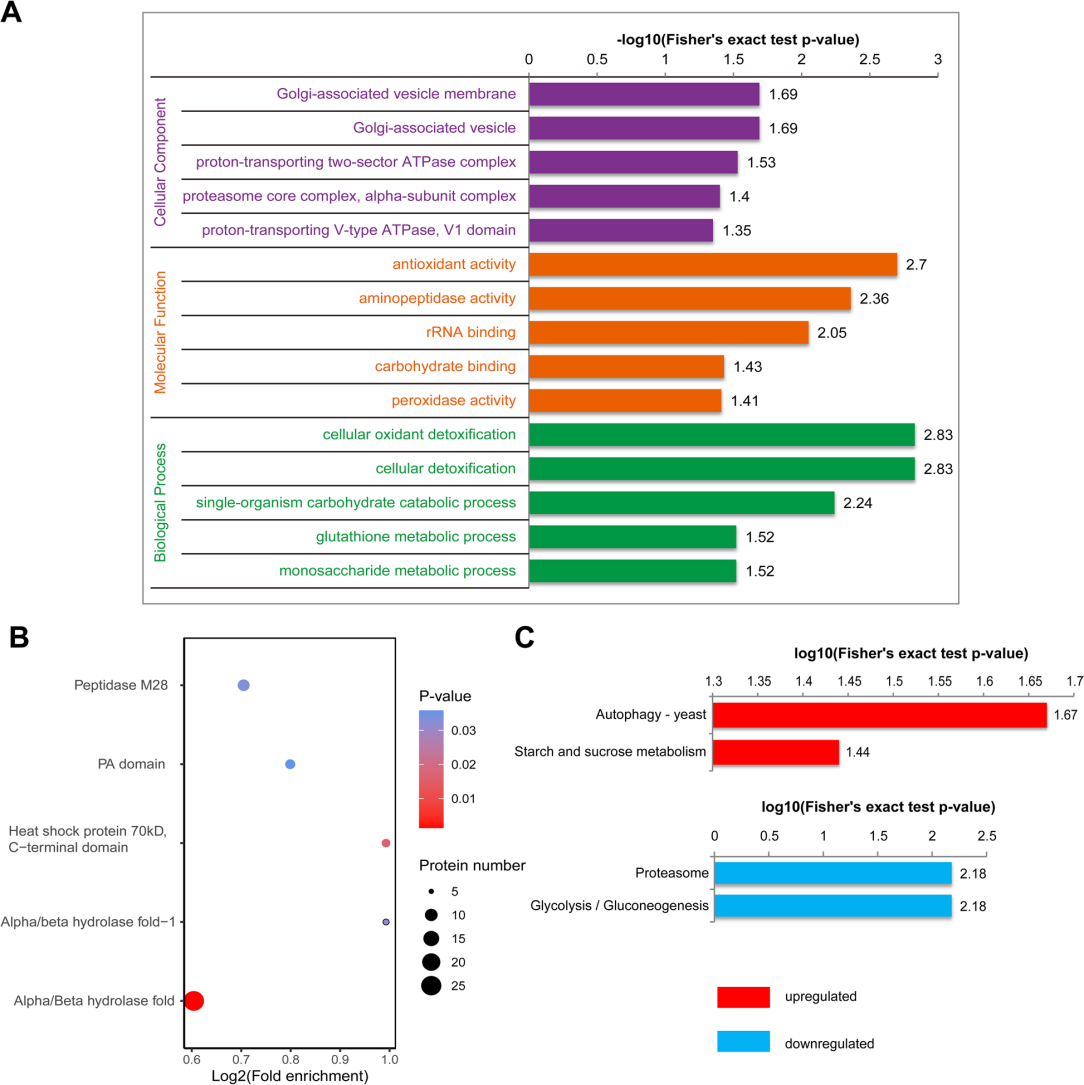
Enrichment analysis of TAKPs in *F. graminearum*. **A** GO enrichment of TAKPs. -Log10(Fisher’s exact p-value) is shown on the x-axis. A GO term with a *p*-value < 0.05 was considered significant. Detailed data are listed in Additional File 7: Table S7. **B** Protein domain enrichment analysis of TAKPs. Detailed data are listed in Additional File 8: Table S8. **C** KEGG pathway enrichment analysis of TAKPs. Detailed data are listed in Additional File 9: Table S9. In all the enrichment analyses, a *p*-value < 0.05 was considered significant

Then, GO and protein domain classification and enrichment analysis of all the identified TAKPs were conducted. As shown in Fig. 7C, regarding biological processes, 304 TAKPs were involved in metabolic processes, 200 in cellular processes and 187 in single-organism processes. In the cellular component category, 169 TAKPs were cell proteins, 119 were organelle proteins, 92 were membrane proteins and 88 were macromolecular complex proteins. Regarding molecular function, 287 proteins were involved in catalytic activity, 190 proteins in binding activity and 35 in structural molecule activity. We did not determine the preference of TAKPs compared to the global Khib proteins in the classification analysis. Moreover, the biological process enrichment analysis of TAKPs, especially the upregulated proteins, indicated that the most significantly enriched terms were related to cellular detoxification; molecular function enrichment also showed that antioxidant activity, aminopeptidase activity, and peroxidase activity were among the enriched terms; and in the cellular component category, Golgi-associated vesicle, proton-transporting ATPase complex, and proteasome core complex-related components were significantly enriched (Fig. 8A, Additional File 10: Figure S3, and Additional File 7: Table S7). All these GO terms were associated with the stress response, which demonstrated that 2-hydroxyisobutyrylation was reshaped in response to fungicide treatment. This assumption was also supported by the analyses of protein domain enrichment and KEGG pathway enrichment (Fig. 8B and 8C; Additional File 10: Figure S4, and Additional File 8–9: Table S8 and Table S9), where the related domains of the hydrolase fold, peptidase M26 and heat shock protein and the KEGG pathway phagosome (fgr04145) were significantly enriched among all the Khib proteins. The KEGG pathways autophagy (fgr04138), starch and sucrose metabolism (fgr00500), proteasome (fgr03050), and glycolysis/gluconeogenesis (fgr00010) were also significantly enriched in the analysis of upregulated and downregulated Khib proteins.

### Khib proteins involved in virulence and DON production in *F. graminearum*

*F. graminearum* is the predominant causal agent of wheat scab disease and produces several mycotoxins, including DON. DON not only threatens the health of livestock and humans but also acts as a critical virulence factor in the fungal infection process. In our data, we found that many proteins involved in pathogenicity were identified to be 2-hydroxyisobutyrylated (Table 2). These proteins are involved in several biological processes, including transcription, translation, and autophagy. For example, FgAtg8 and FgAtg15 function in the autophagy pathway, and their deletion mutants showed reduced conidium production, reduced DON production and impaired virulence compared with the wild type [28, 29]. FgPrb1 is a subtilisin protease and is involved in autophagy regulation. Deletion of FgPrb1 also led to decreased DON production and pathogenicity [30]. Three components of the retromer complex, namely, FgVps5, FgVps29 and FgVps35, were also identified in the Khib list. Loss of any gene among them could lead to growth and development defects in *F. graminearum* [31]. An increasing number of reports have shown that protein PTMs could play critical roles in fungal virulence. For example, acetylation of the autophagy-related proteins MoAtg3 and MoAtg9 by the histone acetyltransferase MoHat1 is important for autophagy and pathogenicity in *Magnaporthe oryzae* [32]. Acetylation of the BcHpt K161 site can affect virulence in *Botrytis cinerea* [33]. A recent study revealed that both lysine succinylation and SUMOylation play roles in virulence and aflatoxin biosynthesis in *Aspergillus flavus* [34, 35]. In recent reports, Khib in two plant pathogenic fungi, *U. virens* and *B. cinerea*, was found to be involved in fungal virulence [8, 9]. Here, our data also provide extensive insights to develop the possible link between Khib modification and fungal virulence and DON production in *F. graminearum*.

**Table 2:** List of Khib proteins involved in DON production and virulence in *F. graminearum*

Protein	Gene locus	Predicted functions	DON production	Virulence	Khib influenced by TEC or not	References
Ptc3	FGSG_10239	Phosphatase	reduced	reduced	no	[36]
FgHal2	FGSG_09532	Phosphatase	loss	reduced	yes	[37, 38]
FgMcm1	FGSG_08696	MADS-box transcription factor	reduced	reduced	no	[39]
GzMyb016	FGSG_10269	Myb transcription factor	loss	normal	no	[40]
FgAtg8	FGSG_10740	Component of autophagosomes and Cvt vesicles	reduced	reduced	yes	[41]
FgAtg15	FGSG_02519	Lipase required for intravacuolar lysis of autophagic and Cvt bodies	reduced	reduced	yes	[28]
FgVps5	FGSG_02011	A subunit of retromer complex, vacuolar protein sorting-associated	reduced	reduced	no	[31]
FgVps35	FGSG_02756	A subunit of retromer complex, vacuolar protein sorting-associated	reduced	reduced	yes	[31]
FgVps29	FGSG_01552	A subunit of retromer complex, vacuolar protein sorting-associated	reduced	reduced	no	[31]
FgChs5	FGSG_01964	Chitin synthase	reduced	loss	no	[42, 43]
FgGpa1	FGSG_05535	Putative Ga subunit	Increased	normal	no	[44]
FgCap1	FGSG_01923	Adenylate cyclase-associated protein related to cAMP signaling	reduced	reduced	no	[45]
Acl2	FGSG_06039	ATP citrate lyase	reduced	reduced	yes	[46]
Fim	FGSG_09862	Fimbrin	reduced	reduced	yes	[46]
FgIlv5	FGSG_10118	Keto-acid reductoisomerase	reduced	reduced	no	[47]
FgGlx	FGSG_11097	Glyoxal oxidase	reduced	reduced	yes	[48]
FgSrp1	FGSG_09864	SR (serine/arginine-rich) proteins in pre-mRNA splicing and processing	reduced	reduced	yes	[49]
FgEb1	FGSG_06627	Microtubule end-binding protein	reduced	reduced	no	[50]
OXP1	FGSG_10203	5-oxoprolinase	reduced	reduced	yes	[51]
FgZuo	FGSG_02785	Hsp70 cochaperone	reduced	reduced	no	[52]
FgSsz	FGSG_08644	Hsp70 protein	reduced	reduced	no	[52]
FgPrb1	FGSG_00192	Subtilisin-like protease	reduced	reduced	yes	[30]

### Enzymes involved in sterol biosynthesis are modified on Khib sites by tebuconazole

TEC, an azole fungicide, works as an inhibitor targeting sterol 14-demethylase, encoded by *CYP51B* and *CYP51A*, to block the sterol biosynthesis that is essential for fungal growth in *F. graminearum* [53]. The specific targeting led us to examine all the sterol biosynthesis enzymes that harbored the Khib site and the possibility of modulating Khib activity by using TEC. Typically, fungal sterol biosynthesis consists of two stages according to KEGG pathway analysis. The terpenoid backbone is synthesized in the first stage (fgr00900), while steroid biosynthesis is synthesized in the second stage (fgr00100). Four enzymes, in the first stage, were identified to be 2-hydroxyisobutyrylated, and all the enzymes played roles in the biosynthesis pathways of farnesyl pyrophosphate (farnesyl-PP) (Table 3, Additional File 10: Figure S5). Three of the four, except FGSG_05911, showed enhanced Khib modification after TEC treatment. Remarkably, the farnesyl diphosphate synthase FGSG_06784 harbored nine Khib sites, and five of them were upregulated at the level of Khib by TEC (Table 3). In the second stage of sterol biosynthesis, 16 Khib sites were identified in eight proteins, and three sites from three proteins were remodified at Khib sites by TEC. Specifically, there was a notable suppression, approximately 3.24-fold, of the Khib level on the only identified Khib site in ERG6B (FGSG_02783) by TEC. In addition, two Khib sites on FgERG9 (FGSG_09381) were identified, and the Khib modification was enhanced at one site, K381, after TEC treatment. Six Khib sites from FgCYP51B (FGSG_01000) were identified, and the Khib on one site was suppressed to a lower level by TEC.

**Table 3:** List of Khib proteins involved in sterol biosynthesis in *F. graminearum*

Gene locus	Protein accession	Gene function	Number of Khib sites	Number of changed Khib sites^a^
**Khib proteins involved in terpenoid backbone biosynthesis (fgr00900)**				
FGSG_10424	I1S130	MVD, ERG19, diphosphomevalonate decarboxylase [EC:4.1.1.33]	2	1 up
FGSG_09722	I1RZ92	IDI; isopentenyl-diphosphate delta-isomerase [EC:5.3.3.2]	4	1 up
FGSG_06784	V6RG22	FDPS; farnesyl diphosphate synthase [EC:2.5.1.1 2.5.1.10]	9	5 up
FGSG_05911	A0A098E2N1	FNTA; protein Farnesyltransferase/geranylgeranyltransferase type-1 subunit alpha [EC:2.5.1.58 2.5.1.59]	1	0
**Khib proteins involved in steroid biosynthesis**				
FGSG_09381	V6RPN0	FDFT1, ERG9; farnesyl-diphosphate farnesyltransferase [EC:2.5.1.21]	2	1 up
FGSG_04092	I1RJR2	CYP51A; sterol 14-demethylase [EC:1.14.13.70]	1	0
FGSG_01000	I1RBR4	CYP51B; sterol 14-demethylase [EC:1.14.13.70]	6	1 down
FGSG_01203	I1RCA0	NSDHL, ERG26; sterol-4alpha-carboxylate 3-dehydrogenase (decarboxylating) [EC:1.1.1.170]	2	0
FGRAMPH1_ 01T26961	A0A1C3YLL7	ERG27; 3-keto steroid reductase [EC:1.1.1.270]	1	0
FGSG_05740	A0A0E0SMA3	SMT1A, ERG6A; sterol 24-C-methyltransferase [EC:2.1.1.41]	1	0
FGSG_02783	I1RGC4	SMT1B, ERG6B; sterol 24-C-methyltransferase [EC:2.1.1.41]	1	1 down
FGSG_07315	I1RT23	ERG2; C-8 sterol isomerase [EC:5.-.-.-]	2	0

^a^ Number of Khib sites which influenced by TEC

In addition, we also examined the Khib modification in the regulatory transcription factors involved in sterol biosynthesis in fungi. There are two theories regarding the regulatory mechanisms of sterol biosynthesis. One involves the sterol regulatory element-binding protein (SREPBP), which is found in mammals and some fungi, such as *Aspergillus fumigatus*, *Cryptococcus neoformans*, and *Schizosaccharomyces pombe* [54–56]. The other is the transcription factor Upc2 in *Saccharomycotina cerevisiae* [57]. However, Liu et al. recently reported that sterol biosynthesis in *F. graminearum* was not regulated by SREBP or Upc2 orthologs but was directed by the phosphorylated transcription factor FgSR [58]. However, our results revealed that there were no Khib sites identified in SREBP orthologs, Upc2 orthologs or FgSR.

**Fig. 9:**
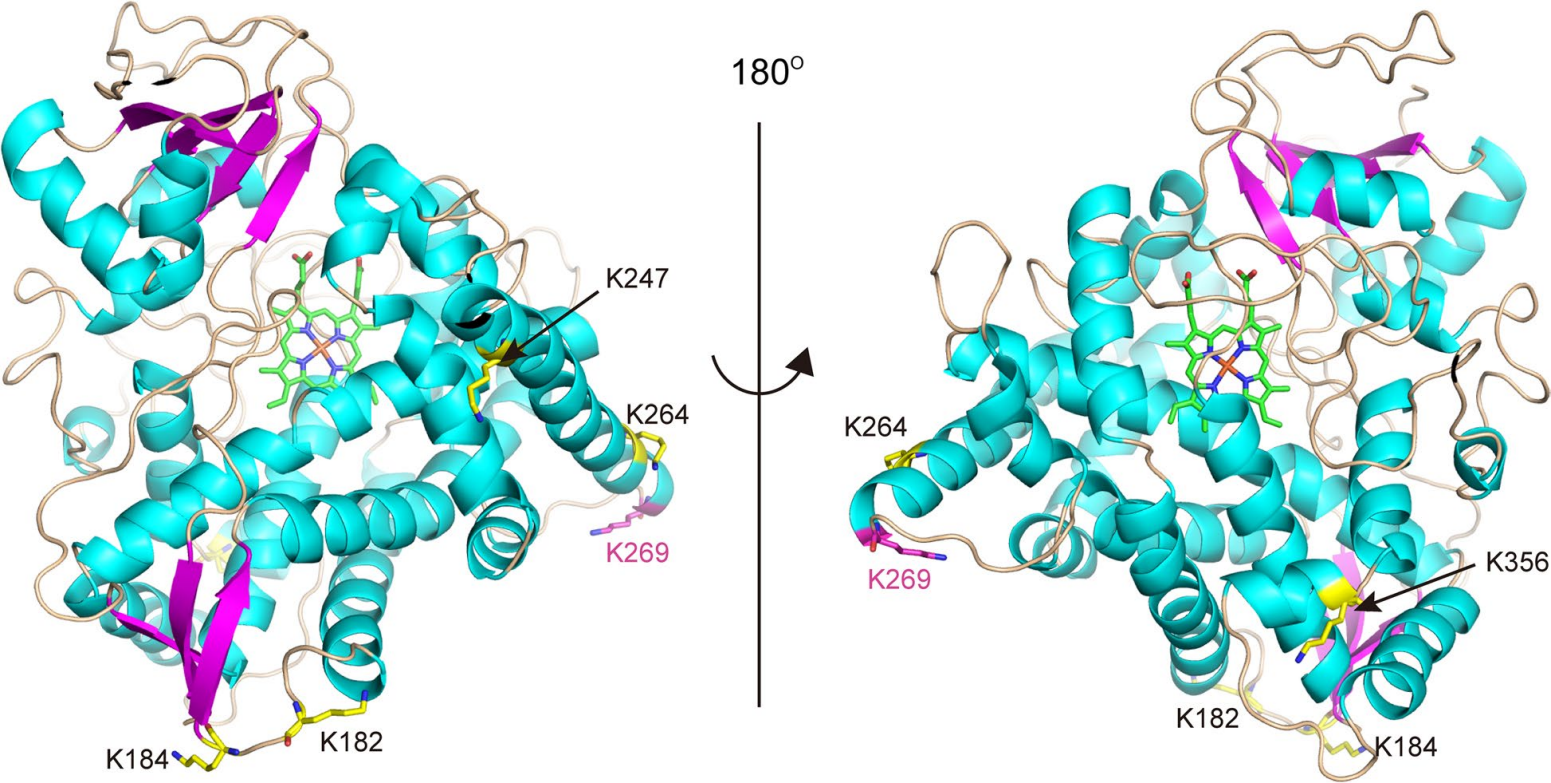
Illustration of the predicted FgCYP51B structure reported by Qian et al. [61]. The heme molecule and Khib sites are shown as sticks, and the positions are labeled. Carbon atoms of the suppressed Khib site K269 are colored in magenta, and those of all the other five Khib sites are colored in yellow. The figure was prepared using PyMOL (v2.4.1, Schrödinger, Inc.)

Our results revealed that the status of Khib in the sterol biosynthesis pathways in *F. graminearum* was reshaped by TEC. In the first stage, the stressed fungi accumulated higher levels of Khib modification on three enzymes that were implicated in the biosynthesis of farnesyl-PP, which is an intermediate metabolite for steroids and is associated with many biological processes, such as protein modification [59] and the biosynthesis of various secondary metabolites, including sterol and DON [53, 60]. In the second stage, TEC led to an elevated Khib level on the farnesyl-diphosphate farnesyltransferase ERG9, which directed the conversion of farnesyl-PP to squalene. The suppression of Khib on ERG6B, a sterol 24-C-methyltransferase, ranking as the second strongest effect, was notable given that a recent study showed that *FgERG6B* was regulated by FgSR, the master regulator of sterol biosynthesis in *F. graminearum* [58]. In addition, the suppression of Khib on one site of FgCYP51B is also notable. Based on the structural interaction between TEC and FgCYP51B [61], the suppressed Khib site seems to be far away from the ligand-binding pocket (Fig. 9). It is unknown how the physical interaction between TEC and FgCYP51B attenuates the Khib level on FgCYP51B.

Two studies revealed that succinylation and malonylation on lysine residues changed enzyme activity [62, 63]. Did the change in Khib status on the listed proteins involved in sterol biosynthesis tell a similar story? Further experiments are required to reveal the potential role of these remodified Khib sites with respect to azole fungicide sensitivity.

## Conclusions

In this study, we performed a TMT-based quantitative lysine 2-hydroxyisobutyrylomic analysis using high-resolution LC–MS/MS in combination with high-specific affinity enrichment. We identified 3501 Khib sites on 1049 proteins in *F. graminearum*. Among them, 3035 Khib sites on 937 proteins were quantifiable, and 1083 Khib sites on 556 modified proteins were affected significantly by TEC treatment. Our results revealed that Khib proteins were localized to multiple cellular compartments, involved in a wide range of biological processes, and associated with virulence and DON production, as well as sterol biosynthesis, in *F. graminearum*. Most Khib proteins affected by TEC were upregulated, and the enrichment analysis showed that TAKPs were significantly enriched in terms associated with the stress response. Specifically, several Khib-modified enzymes in sterol biosynthesis were affected by tebuconazole. These results expanded our understanding of the biological functions of Khib in *F. graminearum*. This study also offers a wealth of resources for further study of the roles of Khib in the fungicide resistance of *F. graminearum*.

## Methods

### Fungal strain and culture

The *F. graminearum* strain PH-1, stored in the key lab of integrated crop disease and pest management of Shandong Province (China), was used in this study and cultured on potato dextrose agar (PDA) at 25 °C. Conidia were induced and collected according to a previous report [53]. A total of 2 × 10^5^ spores were cultured in potato dextrose broth (PDB) at 25 °C for 72 h. Then, TEC (dissolved in dimethyl sulfoxide (DMSO)) was added at a final concentration of 0.09 μg/mL, which is close to the previously reported EC50 value [64]. In the control sample, the same amount of DMSO was added. Mycelia were collected by centrifugation after further incubation for 24 h.

### Protein extraction

The harvested mycelia were first ground to powder in liquid nitrogen. After the addition of four volumes of lysis buffer (1% Triton X-100, 10 mM dithiothreitol (DTT), 1% protease inhibitor cocktail, 3 μM trichostatin A (TSA), 50 mM nicotinamide (NAM)), samples were lysed by sonication. An equal volume of Tris-saturated phenol (pH 8.0) was added to the lysate, and then the mixture was further vortexed. After centrifugation at 4 °C and 5 000 × g for 10 min, the upper solution was transferred to a new centrifuge tube. Proteins were precipitated by adding at least four volumes of ammonium sulfate-saturated methanol and incubating at -20 °C overnight. Then, the samples were centrifuged at 4 °C for 10 min, and the resulting pellets were washed once with cold acetone and then washed three times with cold acetone. Finally, the protein was redissolved in 8 M urea, and the protein concentration was determined with a BCA kit (Beyotime, China) according to the manufacturer’s instructions. For each treatment, protein extracted from three independent biological replicates was mixed for the subsequent assays.

### Western blot

The proteins were extracted as mentioned above. For each sample, 15 μg of proteins was separated by 12% SDS-PAGE and then transferred to a polyvinylidene fluoride (PVDF) membrane. The membrane was then blocked with 5% milk. Then, the total Khib proteins were detected with a pan anti-2-hydroxyisobutyryllysine antibody (PTM BioLabs, China).

### Trypsin digestion, TMT labeling and affinity enrichment

Before trypsin digestion, the protein solution was first reduced and alkylated and then diluted with triethylammonium bicarbonate (TEAB) to reduce the concentration of urea to less than 2 M. Finally, the proteins were digested with trypsin (Promega, USA) at a trypsin-to-protein mass ratio of 1:50 for the first digestion overnight and 1:100 for a second 4 h digestion. Then, the tryptic peptides were desalted with a Strata X C18 SPC column (Phenomenex, USA), vacuum dried, and reconstituted in 0.5 M TEAB. The sample was subsequently processed according to the manufacturer’s protocol for a tandem mass tag (TMT) kit (Thermo Fisher, USA). Briefly, one unit of TMT reagent was thawed and dissolved in acetonitrile. After incubation for 2 h, the reaction was stopped with hydroxylamine. The peptide mixtures were then pooled, desalted, and dried. Then, the tryptic peptides were dissolved in NETN buffer (100 mM NaCl, 1 mM EDTA, 50 mM Tris–HCl, 0.5% NP-40; pH 8.0) and incubated with prewashed anti-Khib antibody beads (PTM Bio, China) at 4 °C overnight with gentle shaking. Then, the bound peptides were eluted from the beads with 0.1% trifluoroacetic acid, followed by four washes with NETN buffer and two washes with H_2_O. The resulting peptides were desalted with C18 ZipTips (Millipore, USA) according to the manufacturer’s instructions.

### UPLC-MS/MS analysis

UPLC separation was performed on an in-house reversed-phase analytical column (15-cm length, 75 μm i.d.) using an EASY-nLC 1000 UPLC system. The tryptic peptides were first dissolved in 0.1% formic acid. The mobile phase, composed of solvent A (0.1% formic acid in 2% acetonitrile) and solvent B (0.1% formic acid in 98% acetonitrile), was pumped at a flow rate of 350 nL/min. The elution gradient was set as follows: 0–38 min, 10%-25% solvent B; 38–52 min, 25%-38% solvent B; 52–56 min, 38%-80% solvent B; and holding at 80% solvent B for at least 3 min.

The peptides were then subjected to nanoelectrospray ionization (NSI) followed by tandem mass spectrometry (MS/MS) in a Q Exactive™ Plus instrument (Thermo Fisher, USA) coupled online to the UPLC instrument. The electrospray voltage applied was 2.0 kV. The intact peptides and ion fragments were detected in the Orbitrap at resolutions of 70 000 and 17 500, respectively. The full MS scan range was set from 350 m/z to 1800 m/z. Peptides were then selected for MS/MS using normalized collision energy (NCE) setting of 28. The fixed first mass was set to 100 m/z. A data-dependent acquisition procedure that alternated between one MS scan followed by 20 MS/MS scans was applied to the top 20 precursor ions with the highest signal intensity with 15.0 s dynamic exclusion. Automatic gain control (AGC) was set at 5E4 with a maximus ion injection time of 200 ms.

### Database search

The MaxQuant search engine (v.1.5.8) was used to process the raw MS/MS data [65, 66]. Tandem mass spectra were searched against the UniProt *Gibberella zeae* strain PH-1 protein database (version 2019.06, 14,160 entries) concatenated with the reverse decoy database. Trypsin/P was specified as the cleavage enzyme with up to 2 missing cleavages allowed and 5 modifications per peptide. The mass tolerance for precursor ions and fragment ions was set as 20 ppm and 0.02 Da, respectively. Carbamidomethylation on cysteine was specified as a fixed modification, and N-terminal acetylation, decarboxamidation, oxidation on methionine and lysine 2-hydroxyisobutyrylation were specified as variable modifications. The false discovery rate (FDR) was adjusted to < 0.01, and the minimum score for modified peptides was set to > 40. Several representative MS/MS spectra and peak assignments for the Khib peptides are shown in Additional File 10: Figure S6.

### Bioinformatics methods

Gene Ontology (GO) annotations of the 2-hydroxyisobutyrylome were derived from the UniProt-GOA database (http://www.ebi.ac.uk/GOA/) [67]. All the Khib proteins were classified by GO annotation based on three categories: biological process, cellular component, and molecular function [68, 69]. The Kyoto Encyclopedia of Genes and Genomes (KEGG) database was used to annotate protein pathways. The KEGG pathways of all identified Khib proteins were annotated using the KEGG online service tools KAAS (https://www.genome.jp/tools/kaas/) and KEGG Mapper (https://www.genome.jp/kegg/mapper.html) [70, 71]. Protein domain annotation was performed by InterProScan based on the protein sequence alignment method, and the InterPro domain database was used [72]. The program WoLF PSORT, an updated version of PSORT/PSORT II, was used for subcellular localization prediction [73]. MoMo software (Motif-x algorithm) was employed to analyze the model of sequences constituted by amino acids in specific positions of modify-21-mers (10 amino acids upstream and downstream of the Khib site) in all protein sequences [74].

For GO, KEGG pathway and protein domain enrichment analyses, a Perl module (https://metacpan.org/pod/Text::NSP::Measures::2D::Fisher) was used to perform a two-tailed Fisher’s exact test to verify the enrichment of Khib proteins against all identified proteins. In all analyses, a term with a corrected *p*-value < 0.05 was considered significant. A protein–protein interaction network of the Khib proteins was generated by STRING by searching against the STRING database, version 10.1, and visualized by the R package “networkD3” (https://CRAN.R-project.org/package=networkD3) [75]. All interactions with a confidence score ≥ 0.7 (high confidence) were fetched.

## Supplementary Information

**Additional file 1: Table S1.** The detailed MS information of quantifiable Khib sites and quantifiable peptides in *F. graminearum*.

**Additional file 2: Table S2**. Conserved Khib sites and orthologous proteins of *F. graminearum* identified in *Candida albicans*,* Fusarium oxysporum*, *Botrytis cinerea*, and* Ustilaginoidea virens.*

**Additional file 3: Table S3**. The GO enrichment analysis of all the identified Khib proteins in *F. graminearum*.

**Additional file 4: Table S4**. The protein domain enrichment analysis of all the identified Khib proteins in* F. graminearum*.

**Additional file 5: Table S5**. The KEGG pathway enrichment of all the identified Khib proteins in* F. graminearum*.

**Additional file 6: Table S6**. The identified tebuconazole-affected Khib sites in *F. graminearum*.

**Additional file 7: Table S7**. The GO enrichment analysis of tebuconazole-affected Khib proteins (TAKPs) in *F. graminearum*.

**Additional file 8: Table S8**. The protein domain enrichment analysis of tebuconazole-affected Khib proteins (TAKPs) in *F. graminearum*.

**Additional file 9: Table S9**. The KEGG pathway enrichment analysis of tebuconazole-affected Khib proteins (TAKPs) in* F. graminearum*.

**Additional file 10: Figure S1.** The GO enrichment analysis of all the identified proteins in *F. graminearum.* -Log10(Fisher’s exact p-value) is shown as the x-axis. A GO term with a p-value < 0.05 was considered significant. Detailed data are listed in Additional File 3: Table S3. **Figure S2.** Protein-protein interaction network of all the identified Khib proteins in *F. graminearum*, built against the STRING database (version 10.1). Identified interactions with confidence score ≥ 0.7 (high confidence) were fetched and visualized in R package “networkD3”. **Figure S3**. The GO enrichment analyses of upregulated (A) and downregulated (B) Khib proteins after tebuconazole treatment in *F. graminearum*. -Log10(Fisher’s exact p-value) is shown as the x-axis. A GO term with a p-value < 0.05 is considered significant. Detailed data are listed in Additional File 7: Table S7. **Figure S4**. The protein domain enrichment analyses of upregulated (A) and downregulated (B) Khib proteins after tebuconazole treatment in *F. graminearum*. Detailed data are listed in Additional File 8: Table S8.** Figure S5**. The sterol biosynthesis pathway based on the KEGG pathway fgr00900 and fgr00100, and the report of Fan et al. [53]. All the enzymes with identified Khib sites are labeled in bold, and the corresponding gene locus is indicated on yellow background. Enzyme names with enhanced Khib sites in response to tebuconazole are in red, while those with suppressed Khib sites are in blue. Khib proteins not affected by tebuconazole treatment are indicated in black. **Figure S6**. Several representative MS/MS spectra and peak assignments for the Khib peptides.

## Data Availability

The mass spectrometry-based proteomics data have been deposited to the ProteomeXchange Consortium (http://www.proteomexchange.org/) via the PRIDE partner repository with the dataset accession number: PXD022499. All the other data generated or analyzed during this study are included in this published article and its supplementary information files.

## Abbreviations

Khib: Lysine 2-hydroxyisobutyrylation
PTM: Post-translational modification
LC–MS/MS: Liquid chromatography coupled to tandem mass spectrometry
DON: Deoxynivalenol
EC50: Half-maximal effective concentration
DTT: Dithiothreitol
TSA: Trichostatin A
NAM: Nicotinamide
TEAB: Triethylammonium bicarbonate
NSI: Nano electrospray ionization
NCE: Normalized collision energy
AGC: Automatic gain control
GO: Gene Ontology
KEGG: Kyoto Encyclopedia of Genes and Genomes
FDR: False discovery rate
PPIs: Protein–protein interactions
TEC: Tebuconazole
BCM: Carbendazim
TAKP: Tebuconazole affected Khib proteins
SREPBP: Sterol regulatory element-binding protein

## References

[1] Dai L, Peng C, Montellier E, Lu Z, Chen Y, Ishii H (2014). Lysine 2-hydroxyisobutyrylation is a widely distributed active histone mark. Nat Chem Biol.

[2] Huang J, Luo Z, Ying W, Cao Q, Huang H, Dong J (2017). 2-Hydroxyisobutyrylation on histone H4K8 is regulated by glucose homeostasis in *Saccharomyces cerevisiae*. Proc Natl Acad Sci.

[3] Meng X, Xing S, Perez LM, Peng X, Zhao Q, Redoña ED (2017). Proteome-wide analysis of lysine 2-hydroxyisobutyrylation in developing rice (*Oryza sativa*) seeds. Sci Rep.

[4] Yu Z, Ni J, Sheng W, Wang Z, Wu Y (2017). Proteome-wide identification of lysine 2-hydroxyisobutyrylation reveals conserved and novel histone modifications in *Physcomitrella patens*. Sci Rep.

[5] Dong H, Guo Z, Feng W, Zhang T, Zhai G, Palusiak A (2018). Systematic identification of lysine 2-hydroxyisobutyrylated proteins in *Proteus mirabilis*. Mol Cell Proteomics.

[6] Yin D, Jiang N, Zhang Y, Wang D, Sang X, Feng Y (2019). Global lysine crotonylation and 2-hydroxyisobutyrylation in phenotypically different *Toxoplasma gondii* parasites. Mol Cell Proteomics.

[7] Xue C, Qiao Z, Chen X, Cao P, Liu K, Liu S (2020). Proteome-wide analyses reveal the diverse functions of lysine 2-hydroxyisobutyrylation in *Oryza sativa*. Rice.

[8] Chen X, Li X, Li P, Chen X, Liu H, Huang J (2021). Comprehensive identification of lysine 2-hydroxyisobutyrylated proteins in *Ustilaginoidea virens* reveals the involvement of lysine 2-hydroxyisobutyrylation in fungal virulence. J Integr Plant Biol.

[9] Xu Y, Li X, Liang W, Liu M (2020). Proteome-wide analysis of lysine 2-hydroxyisobutyrylation in the phytopathogenic fungus *Botrytis **cinerea*. Front Microbiol..

[10] Qian H, Wang L, Ma X, Yi X, Wang B, Liang W (2021). Proteome-wide analysis of lysine 2-hydroxyisobutyrylated proteins in *Fusarium **oxysporum*. Front Microbiol..

[11] Zhu W, Jiang X, Sun H, Li Y, Shi W, Zheng M, et al. Global lysine acetylation and 2-hydroxyisobutyrylation reveal the metabolism conversion mechanism in *Giardia lamblia*. Mol Cell Proteomics. 2021;20:100043. 10.1074/mcp.RA120.002353 PMC8724866 33376196

[12] Zheng H, Song N, Zhou X, Mei H, Li D, Li X (2021). Proteome-wide analysis of lysine 2-hydroxyisobutyrylation in *Candida **albicans*. mSystems..

[13] Huang H, Tang S, Ji M, Tang Z, Shimada M, Liu X (2018). p300-mediated lysine 2-hydroxyisobutyrylation regulates glycolysis. Mol Cell.

[14] Chen Y, Kistler HC, Ma Z (2019). *Fusarium graminearum* trichothecene mycotoxins: biosynthesis, regulation, and management. Annu Rev Phytopathol.

[15] Goswami RS, Kistler HC (2004). Heading for disaster: *Fusarium graminearum* on cereal crops. Mol Plant Pathol.

[16] Dean R, Kan Ja LV, Pretorius ZA, Hammond-Kosack KE, Pietro AD, Spanu PD (2012). The top 10 fungal pathogens in molecular plant pathology. Mol Plant Pathol..

[17] Liu X, Jiang J, Shao J, Yin Y, Ma Z (2010). Gene transcription profiling of *Fusarium graminearum* treated with an azole fungicide tebuconazole. Appl Microbiol Biotechnol.

[18] Becher R, Weihmann F, Deising HB, Wirsel SG (2011). Development of a novel multiplex DNA microarray for *Fusarium graminearum* and analysis of azole fungicide responses. BMC Genomics.

[19] Hou Y, Zheng Z, Xu S, Chen C, Zhou M (2013). Proteomic analysis of *Fusarium graminearum* treated by the fungicide JS399-19. Pestic Biochem Physiol.

[20] Omrane S, Sghyer H, Audéon C, Lanen C, Duplaix C, Walker A-S (2015). Fungicide efflux and the MgMFS1 transporter contribute to the multidrug resistance phenotype in *Zymoseptoria tritici* field isolates. Environ Microbiol.

[21] Sang H, Hulvey J, Popko JT, Lopes J, Swaminathan A, Chang T (2015). A pleiotropic drug resistance transporter is involved in reduced sensitivity to multiple fungicide classes in Sclerotinia homoeocarpa (F.T. Bennett). Mol Plant Pathol..

[22] Zhang L, Wang L, Liang Y, Yu J (2019). FgPEX4 is involved in development, pathogenicity, and cell wall integrity in *Fusarium graminearum*. Curr Genet.

[23] Li X, Fan Z, Yan M, Qu J, Xu J-R, Jin Q (2019). Spontaneous mutations in *FgSAD1* suppress the growth defect of the *Fgprp4* mutant by affecting tri-snRNP stability and its docking in *Fusarium graminearum*. Environ Microbiol.

[24] Fernando U, Chatur S, Joshi M, Thomas Bonner C, Fan T, Hubbard K (2019). Redox signalling from NADPH oxidase targets metabolic enzymes and developmental proteins in *Fusarium graminearum*. Mol Plant Pathol.

[25] Lu S, Edwards MC (2016). Genome-wide analysis of small secreted cysteine-rich proteins identifies candidate effector proteins potentially involved in *Fusarium graminearum*−wheat interactions. Phytopathology.

[26] Yu L, He H, Hu Z, Ma Z (2016). Comprehensive quantification of N-glycoproteome in *Fusarium graminearum* reveals intensive glycosylation changes against fungicide. J Proteomics.

[27] Zhou S, Yang Q, Yin C, Liu L, Liang W (2016). Systematic analysis of the lysine acetylome in *Fusarium graminearum*. BMC Genomics.

[28] Nguyen LN, Bormann J, Le GTT, Stärkel C, Olsson S, Nosanchuk JD (2011). Autophagy-related lipase FgATG15 of *Fusarium graminearum* is important for lipid turnover and plant infection. Fungal Genet Biol.

[29] Lv W, Wang C, Yang N, Que Y, Talbot NJ, Wang Z (2017). Genome-wide functional analysis reveals that autophagy is necessary for growth, sporulation, deoxynivalenol production and virulence in *Fusarium graminearum*. Sci Rep.

[30] Xu L, Wang H, Zhang C, Wang J, Chen A, Chen Y (2020). System-wide characterization of subtilases reveals that subtilisin-like protease FgPrb1 of *Fusarium **graminearum* regulates fungal development and virulence. Fungal Genet Biol..

[31] Zheng W, Zheng H, Zhao X, Zhang Y, Xie Q, Lin X (2016). Retrograde trafficking from the endosome to the trans-Golgi network mediated by the retromer is required for fungal development and pathogenicity in *Fusarium graminearum*. New Phytol.

[32] Yin Z, Chen C, Yang J, Feng W, Liu X, Zuo R (2019). Histone acetyltransferase MoHat1 acetylates autophagy-related proteins MoAtg3 and MoAtg9 to orchestrate functional appressorium formation and pathogenicity in *Magnaporthe oryzae*. Autophagy.

[33] Yang Q, Song L, Miao Z, Su M, Liang W, He Y (2020). Acetylation of BcHpt lysine 161 regulates *Botrytis cinerea* sensitivity to fungicides, multistress adaptation and virulence. Front Microbiol.

[34] Nie X, Yu S, Qiu M, Wang X, Wang Y, Bai Y (2016). *Aspergillus flavus* SUMO contributes to fungal virulence and toxin attributes. J Agric Food Chem.

[35] Ren S, Yang M, Yue Y, Ge F, Li Y, Guo X (2018). Lysine succinylation contributes to aflatoxin production and pathogenicity in *Aspergillus flavus*. Mol Cell Proteomics.

[36] Jiang J, Yun Y, Yang Q, Shim W-B, Wang Z, Ma Z (2011). A type 2C protein phosphatase FgPtc3 is involved in cell wall integrity, lipid metabolism, and virulence in *Fusarium **graminearum*. PLOS ONE..

[37] Yu J, Lee K-M, Son M, Kim K-H (2015). Effects of the deletion and over-expression of *Fusarium graminearum* gene *FgHal2* on host response to mycovirus *Fusarium graminearum virus 1*. Mol Plant Pathol.

[38] Yun Y, Liu Z, Yin Y, Jiang J, Chen Y, Xu J-R (2015). Functional analysis of the *Fusarium graminearum* phosphatome. New Phytol.

[39] Yang C, Liu H, Li G, Liu M, Yun Y, Wang C (2015). The MADS-box transcription factor FgMcm1 regulates cell identity and fungal development in *Fusarium graminearum*. Environ Microbiol.

[40] Son H, Seo Y-S, Min K, Park AR, Lee J, Jin J-M (2011). A phenome-based functional analysis of transcription factors in the cereal head blight fungus. Fusarium graminearum. PLoS Pathog..

[41] Josefsen L, Droce A, Sondergaard TE, Sørensen JL, Bormann J, Schäfer W (2012). Autophagy provides nutrients for nonassimilating fungal structures and is necessary for plant colonization but not for infection in the necrotrophic plant pathogen *Fusarium graminearum*. Autophagy.

[42] Kim J-E, Lee H-J, Lee J, Kim KW, Yun S-H, Shim W-B (2009). *Gibberella zeae* chitin synthase genes, *GzCHS5* and *GzCHS7*, are required for hyphal growth, perithecia formation, and pathogenicity. Curr Genet.

[43] Liu Z, Zhang X, Liu X, Fu C, Han X, Yin Y (2016). The chitin synthase FgChs2 and other FgChss co-regulate vegetative development and virulence in F. graminearum. Sci Rep..

[44] Yu H-Y, Seo J-A, Kim J-E, Han K-H, Shim W-B, Yun S-H (2008). Functional analyses of heterotrimeric G protein Gα and Gβ subunits in *Gibberella zeae*. Microbiology.

[45] Yin T, Zhang Q, Wang J, Liu H, Wang C, Xu J-R (2018). The cyclase-associated protein FgCap1 has both protein kinase A-dependent and -independent functions during deoxynivalenol production and plant infection in *Fusarium graminearum*. Mol Plant Pathol.

[46] Sakamoto N, Tsuyuki R, Yoshinari T, Usuma J, Furukawa T, Nagasawa H (2013). Correlation of ATP citrate lyase and acetyl CoA levels with trichothecene production in *Fusarium graminearum*. Toxins.

[47] Liu X, Wang J, Xu J, Shi J (2014). FgIlv5 is required for branched-chain amino acid biosynthesis and full virulence in *Fusarium graminearum*. Microbiology.

[48] Song X-S, Xing S, Li H-P, Zhang J-B, Qu B, Jiang J-H (2016). An antibody that confers plant disease resistance targets a membrane-bound glyoxal oxidase in *Fusarium*. New Phytol.

[49] Zhang Y, Gao X, Sun M, Liu H, Xu J-R (2017). The *FgSRP1* SR-protein gene is important for plant infection and pre-mRNA processing in *Fusarium graminearum*. Environ Microbiol.

[50] Liu Z, Wu S, Chen Y, Han X, Gu Q, Yin Y (2017). The microtubule end-binding protein FgEB1 regulates polar growth and fungicide sensitivity via different interactors in *Fusarium graminearum*. Environ Microbiol.

[51] Yang P, Chen Y, Wu H, Fang W, Liang Q, Zheng Y (2018). The 5-oxoprolinase is required for conidiation, sexual reproduction, virulence and deoxynivalenol production of *Fusarium graminearum*. Curr Genet.

[52] Liu Z, Wang Z, Huang M, Yan L, Ma Z, Yin Y (2017). The FgSsb-FgZuo-FgSsz complex regulates multiple stress responses and mycotoxin production via folding the soluble SNARE Vam7 and β2-tubulin in *Fusarium graminearum*: Functions of the FgSsb-FgZuo-FgSsz complex. Environ Microbiol.

[53] Fan J, Urban M, Parker JE, Brewer HC, Kelly SL, Hammond-Kosack KE (2013). Characterization of the sterol 14α-demethylases of *Fusarium graminearum* identifies a novel genus-specific CYP51 function. New Phytol.

[54] Blatzer M, Barker BM, Willger SD, Beckmann N, Blosser SJ, Cornish EJ (2011). SREBP coordinates iron and ergosterol homeostasis to mediate triazole drug and hypoxia responses in the human fungal pathogen *Aspergillus fumigatus*. PLOS Genet..

[55] Chun CD, Liu OW, Madhani HD (2007). A link between virulence and homeostatic responses to hypoxia during infection by the human fungal pathogen *Cryptococcus **neoformans*. PLOS Pathog..

[56] Hughes AL, Todd BL, Espenshade PJ (2005). SREBP pathway responds to sterols and functions as an oxygen sensor in fission yeast. Cell.

[57] Vik Å, Rine J (2001). Upc2p and Ecm22p, dual regulators of sterol biosynthesis in *Saccharomyces cerevisiae*. Mol Cell Biol.

[58] Liu Z, Jian Y, Chen Y, Kistler HC, He P, Ma Z (2019). A phosphorylated transcription factor regulates sterol biosynthesis in *Fusarium graminearum*. Nat Commun.

[59] Novelli G, D’Apice MR (2012). Protein farnesylation and disease. J Inherit Metab Dis.

[60] Villafana RT, Ramdass AC, Rampersad SN (2019). Selection of *Fusarium* trichothecene toxin genes for molecular detection depends on *TRI* gene cluster organization and gene function. Toxins.

[61] Qian H, Duan M, Sun X, Chi M, Zhao Y, Liang W (2018). The binding mechanism between azoles and FgCYP51B, sterol 14α-demethylase of *Fusarium graminearum*: Interaction between azoles and FgCYP51B. Pest Manag Sci.

[62] Yang M, Wang Y, Chen Y, Cheng Z, Gu J, Deng J (2015). Succinylome analysis reveals the involvement of lysine succinylation in metabolism in pathogenic *Mycobacterium tuberculosis*. Mol Cell Proteomics.

[63] Qian L, Nie L, Chen M, Liu P, Zhu J, Zhai L (2016). Global profiling of protein lysine malonylation in *Escherichia coli* reveals its role in energy metabolism. J Proteome Res.

[64] Qian H, Du J, Chi M, Sun X, Liang W, Huang J (2018). The Y137H mutation in the cytochrome P450 FgCYP51B protein confers reduced sensitivity to tebuconazole in *Fusarium graminearum*. Pest Manag Sci.

[65] Cox J, Mann M (2008). MaxQuant enables high peptide identification rates, individualized p.p.b.-range mass accuracies and proteome-wide protein quantification. Nat Biotechnol..

[66] Tyanova S, Temu T, Cox J (2016). The MaxQuant computational platform for mass spectrometry-based shotgun proteomics. Nat Protoc.

[67] Huntley RP, Sawford T, Mutowo-Meullenet P, Shypitsyna A, Bonilla C, Martin MJ (2015). The GOA database: Gene Ontology annotation updates for 2015. Nucleic Acids Res.

[68] Ashburner M, Ball CA, Blake JA, Botstein D, Butler H, Cherry JM (2000). Gene Ontology: tool for the unification of biology. Nat Genet.

[69] The Gene Ontology Consortium (2019). The Gene Ontology Resource: 20 years and still GOing strong. Nucleic Acids Res.

[70] Kanehisa M, Sato Y (2020). KEGG Mapper for inferring cellular functions from protein sequences. Protein Sci.

[71] Moriya Y, Itoh M, Okuda S, Yoshizawa AC, Kanehisa M (2007). KAAS: an automatic genome annotation and pathway reconstruction server. Nucleic Acids Res..

[72] Mitchell AL, Attwood TK, Babbitt PC, Blum M, Bork P, Bridge A (2019). InterPro in 2019: improving coverage, classification and access to protein sequence annotations. Nucleic Acids Res.

[73] Horton P, Park K-J, Obayashi T, Fujita N, Harada H, Adams-Collier CJ (2007). WoLF PSORT: protein localization predictor. Nucleic Acids Res..

[74] Cheng A, Grant CE, Noble WS, Bailey TL (2019). MoMo: discovery of statistically significant post-translational modification motifs. Bioinformatics.

[75] Szklarczyk D, Gable AL, Lyon D, Junge A, Wyder S, Huerta-Cepas J (2019). STRING v11: protein–protein association networks with increased coverage, supporting functional discovery in genome-wide experimental datasets. Nucleic Acids Res.

