# Supplementary material for: Quantitative multiplexed proteomics analysis reveals reshaping of the lysine 2-hydroxyisobutyrylome in Fusarium graminearum by tebuconazole
Source: BMC Genomics. 2022 Feb 18;23:145. doi: 10.1186/s12864-022-08372-4 (PMC8855566; doi:10.1186/s12864-022-08372-4)
Supplement: Supplementary file 10 — Additional file 10: Figure S1. The GO enrichment analysis of all the identified proteins in F. graminearum. -Log10(Fisher’s exact p-value) is shown as the x-axis. A GO term with a p-value < 0.05 was considered significant. Detailed data are listed in Additional File 3: Table S3. Figure S2. Protein-protein interaction network of all the identified Khib proteins in F. graminearum, built against the STRING database (version 10.1). Identified interactions with confidence score ≥ 0.7 (high confidence) were fetched and visualized in R package “networkD3”. Figure S3. The GO enrichment analyses of upregulated (A) and downregulated (B) Khib proteins after tebuconazole treatment in F. graminearum. -Log10(Fisher’s exact p-value) is shown as the x-axis. A GO term with a p-value < 0.05 is considered significant. Detailed data are listed in Additional File 7: Table S7. Figure S4. The protein domain enrichment analyses of upregulated (A) and downregulated (B) Khib proteins after tebuconazole treatment in F. graminearum. Detailed data are listed in Additional File 8: Table S8. Figure S5. The sterol biosynthesis pathway based on the KEGG pathway fgr00900 and fgr00100, and the report of Fan et al. [53]. All the enzymes with identified Khib sites are labeled in bold, and the corresponding gene locus is indicated on yellow background. Enzyme names with enhanced Khib sites in response to tebuconazole are in red, while those with suppressed Khib sites are in blue. Khib proteins not affected by tebuconazole treatment are indicated in black. Figure S6. Several representative MS/MS spectra and peak assignments for the Khib peptides. [file 12864_2022_8372_MOESM10_ESM.pdf]

Quantitative multiplexed proteomics analysis reveals reshaping of the lysine 2-hydroxyisobutyrylome in *Fusarium graminearum* by tebuconazole

Yanxiang Zhao[1], Limin Zhang[1], Chao Ju[1], Xiaoyan Zhang[2], Jinguang Huang[1*]

[1] College of Plant Health and Medicine and Key Lab of Integrated Crop Disease and Pest Management of Shandong Province, Qingdao Agricultural University, Qingdao 266109, Shandong Province, China

[2] College of Agriculture, Ludong University, Yantai 264025, Shandong Province, China

[*] Corresponding author: jghuang@qau.edu.cn (J.H.)

Supplementary Figures

Figure S1. The GO enrichment analysis of all the identified proteins in *F. graminearum*. -Log10(Fisher's exact p-value) is shown as the x-axis. A GO term with a p-value < 0.05 was considered significant. Detailed data are listed in Additional File 3: Table S3.

Figure S2. Protein-protein interaction network of all the identified Khib proteins in *F. graminearum*, built against the STRING database (version 10.1). Identified interactions with confidence score ≥ 0.7 (high confidence) were fetched and visualized in R package "networkD3".

Figure S3. The GO enrichment analyses of upregulated (A) and downregulated (B) Khib proteins after tebuconazole treatment in *F. graminearum*. -Log10(Fisher's exact p-value) is shown as the x-axis. A GO term with a p-value < 0.05 is considered significant. Detailed data are listed in Additional File 7: Table S7.

Figure S4. The protein domain enrichment analyses of upregulated (A) and downregulated (B) Khib proteins after tebuconazole treatment in *F. graminearum*. Detailed data are listed in Additional File 8: Table S8.

Figure S5. The sterol biosynthesis pathway based on the KEGG pathway fgr00900 and fgr00100, and the report of Fan et al [53]. All the enzymes with identified Khib sites are labeled in bold, and the corresponding gene locus is indicated on yellow background. Enzyme names with enhanced Khib sites in response to tebuconazole are in red, while those with suppressed Khib sites are in blue. Khib proteins not affected by tebuconazole treatment are indicated in black.

Figure S6. Several representative MS/MS spectra and peak assignments for the Khib peptides.

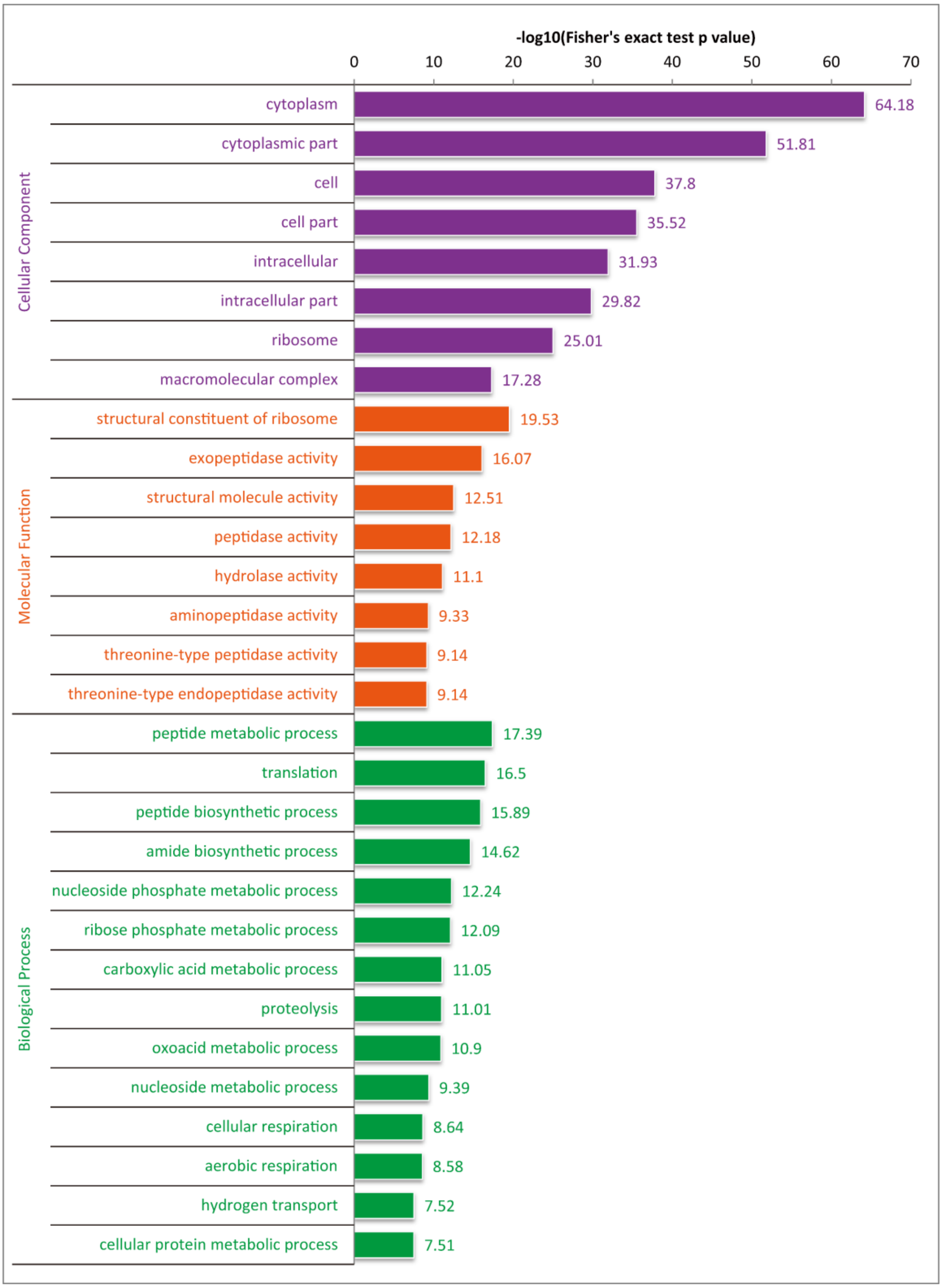

Figure S1. The GO enrichment analysis of all the identified proteins in *F. graminearum*. -Log10(Fisher's exact p-value) is shown as the x-axis. A GO term with a p-value < 0.05 was considered significant. Detailed data are listed in Additional File 3: Table S3.

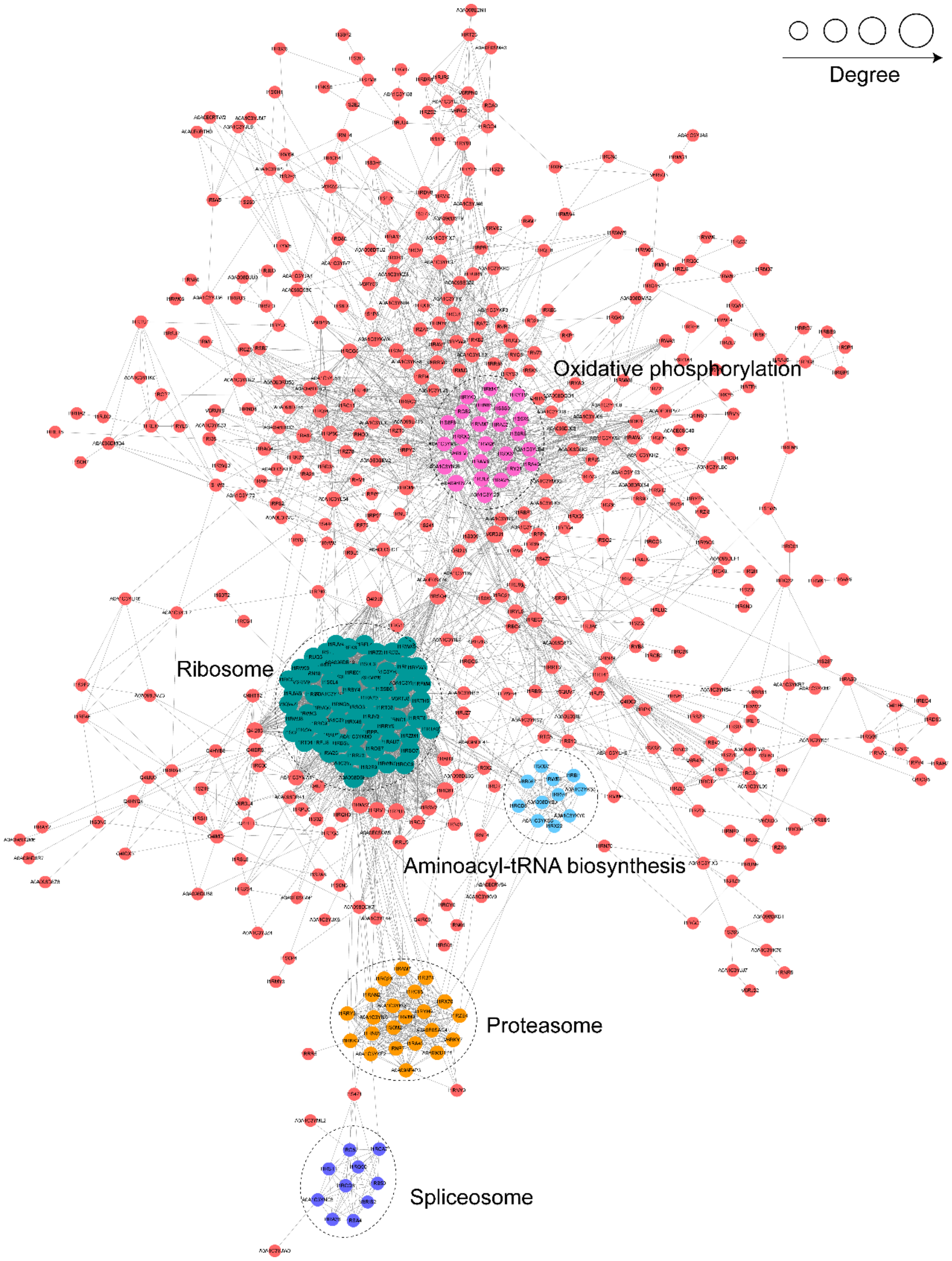

Figure S2. Protein-protein interaction network of all the identified Khib proteins in *F. graminearum*, built against the STRING database (version 10.1). Identified interactions with confidence score ≥ 0.7 (high confidence) were fetched and visualized in R package "networkD3".

A

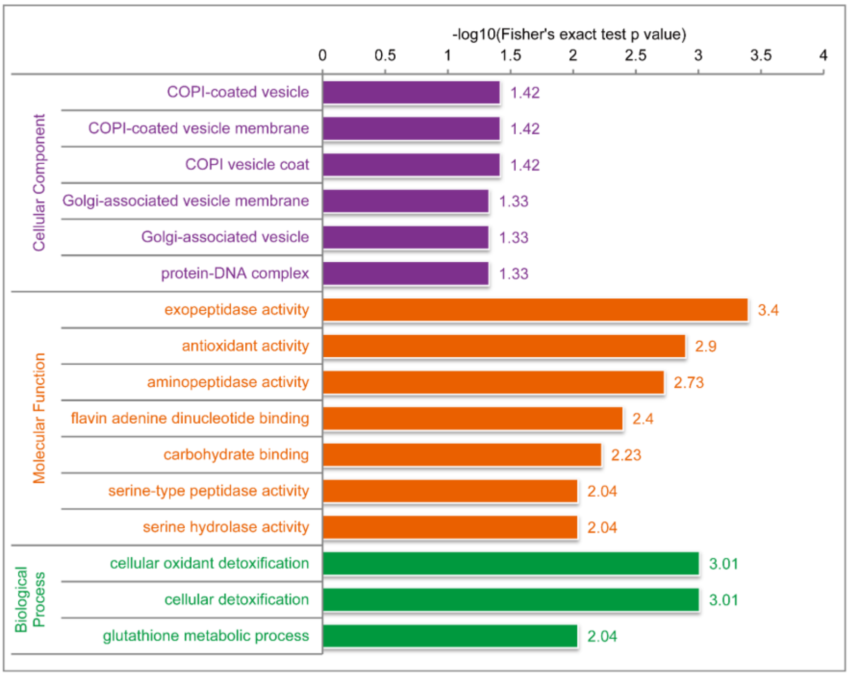

-log10(Fisher's exact test p value)
0
0.5
1
1.5
2
2.5
3
3.5
4
Cellular Component
COPI-coated vesicle 1.42
COPI-coated vesicle membrane 1.42
COPI vesicle coat 1.42
Golgi-associated vesicle membrane 1.33
Golgi-associated vesicle 1.33
protein-DNA complex 1.33
Molecular Function
exopeptidase activity 3.4
antioxidant activity 2.9
aminopeptidase activity 2.73
flavin adenine dinucleotide binding 2.4
carbohydrate binding 2.23
serine-type peptidase activity 2.04
serine hydrolase activity 2.04
Biological Process
cellular oxidant detoxification 3.01
cellular detoxification 3.01
glutathione metabolic process 2.04

B

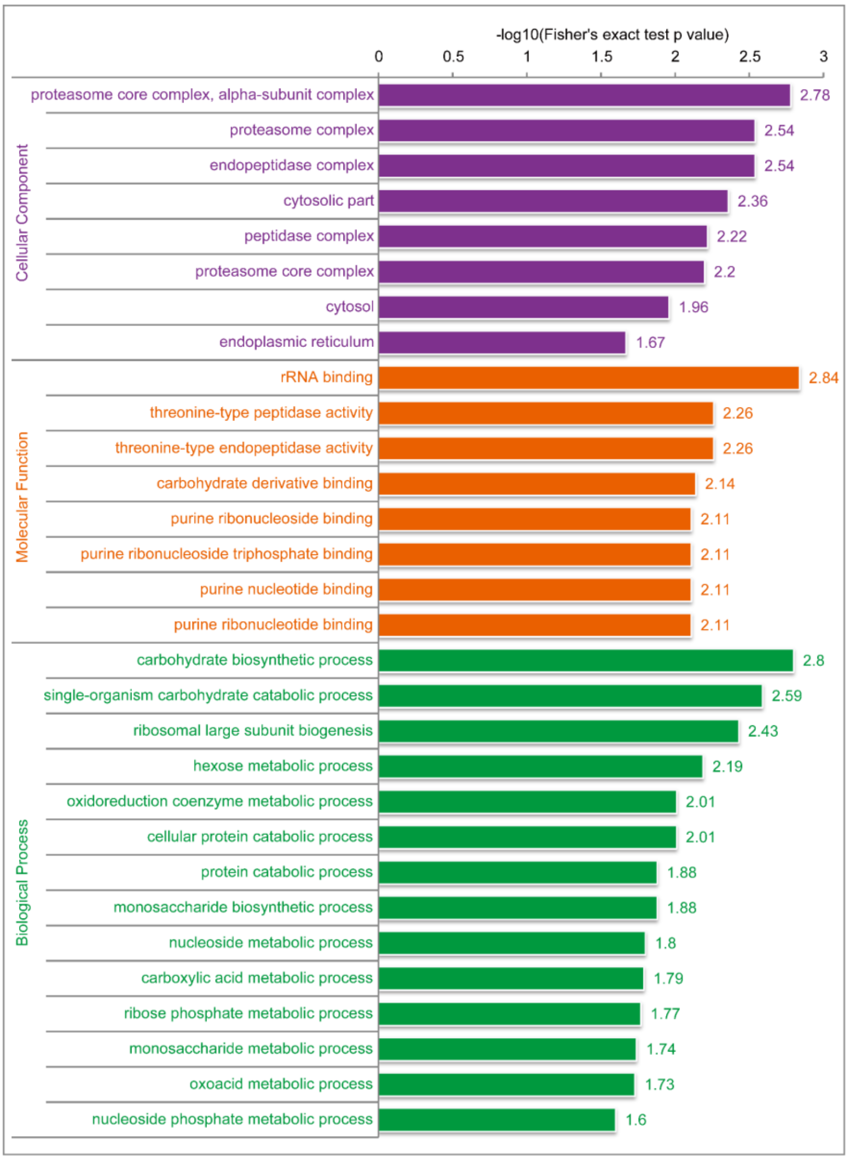

-log10(Fisher's exact test p value)
0
0.5
1
1.5
2
2.5
3
Cellular Component
proteasome core complex, alpha-subunit complex 2.78
proteasome complex 2.54
endopeptidase complex 2.54
cytosolic part 2.36
peptidase complex 2.22
proteasome core complex 2.2
cytosol 1.96
endoplasmic reticulum 1.67
Molecular Function
rRNA binding 2.84
threonine-type peptidase activity 2.26
threonine-type endopeptidase activity 2.26
carbohydrate derivative binding 2.14
purine ribonucleoside binding 2.11
purine ribonucleoside triphosphate binding 2.11
purine nucleotide binding 2.11
purine ribonucleotide binding 2.11
Biological Process
carbohydrate biosynthetic process 2.8
single-organism carbohydrate catabolic process 2.59
ribosomal large subunit biogenesis 2.43
hexose metabolic process 2.19
oxidoreduction coenzyme metabolic process 2.01
cellular protein catabolic process 2.01
protein catabolic process 1.88
monosaccharide biosynthetic process 1.88
nucleoside metabolic process 1.8
carboxylic acid metabolic process 1.79
ribose phosphate metabolic process 1.77
monosaccharide metabolic process 1.74
oxoacid metabolic process 1.73
nucleoside phosphate metabolic process 1.6

Figure S3. The GO enrichment analyses of upregulated (A) and downregulated (B) Khib proteins after tebuconazole treatment in *F. graminearum*. -Log10(Fisher's exact p-value) is shown as the x-axis. A GO term with a p-value < 0.05 is considered significant. Detailed data are listed in Additional File 7: Table S7.

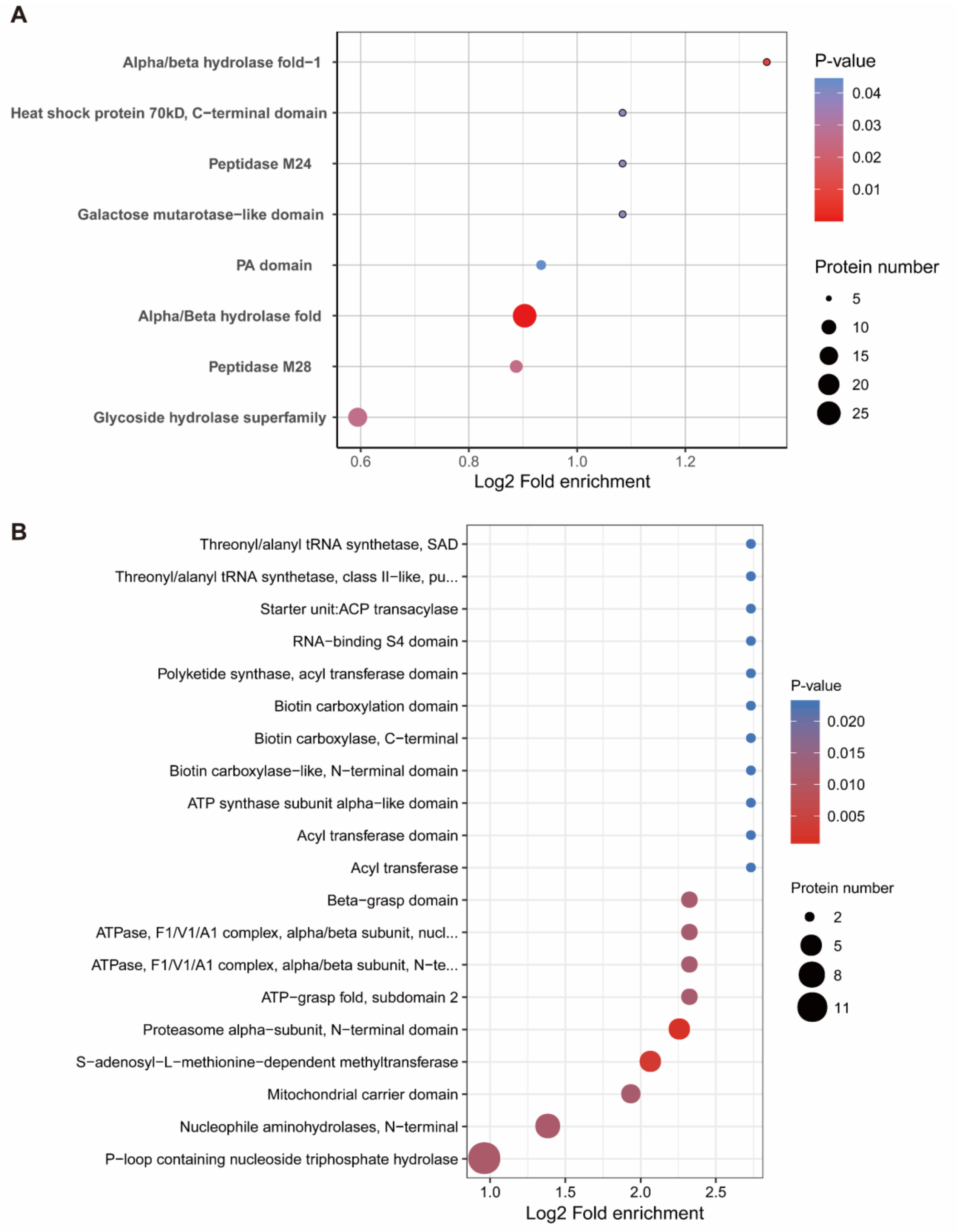

Figure S4. The protein domain enrichment analyses of upregulated (A) and downregulated (B) Khib proteins after tebuconazole treatment in *F. graminearum*. Detailed data are listed in Additional File 8: Table S8.

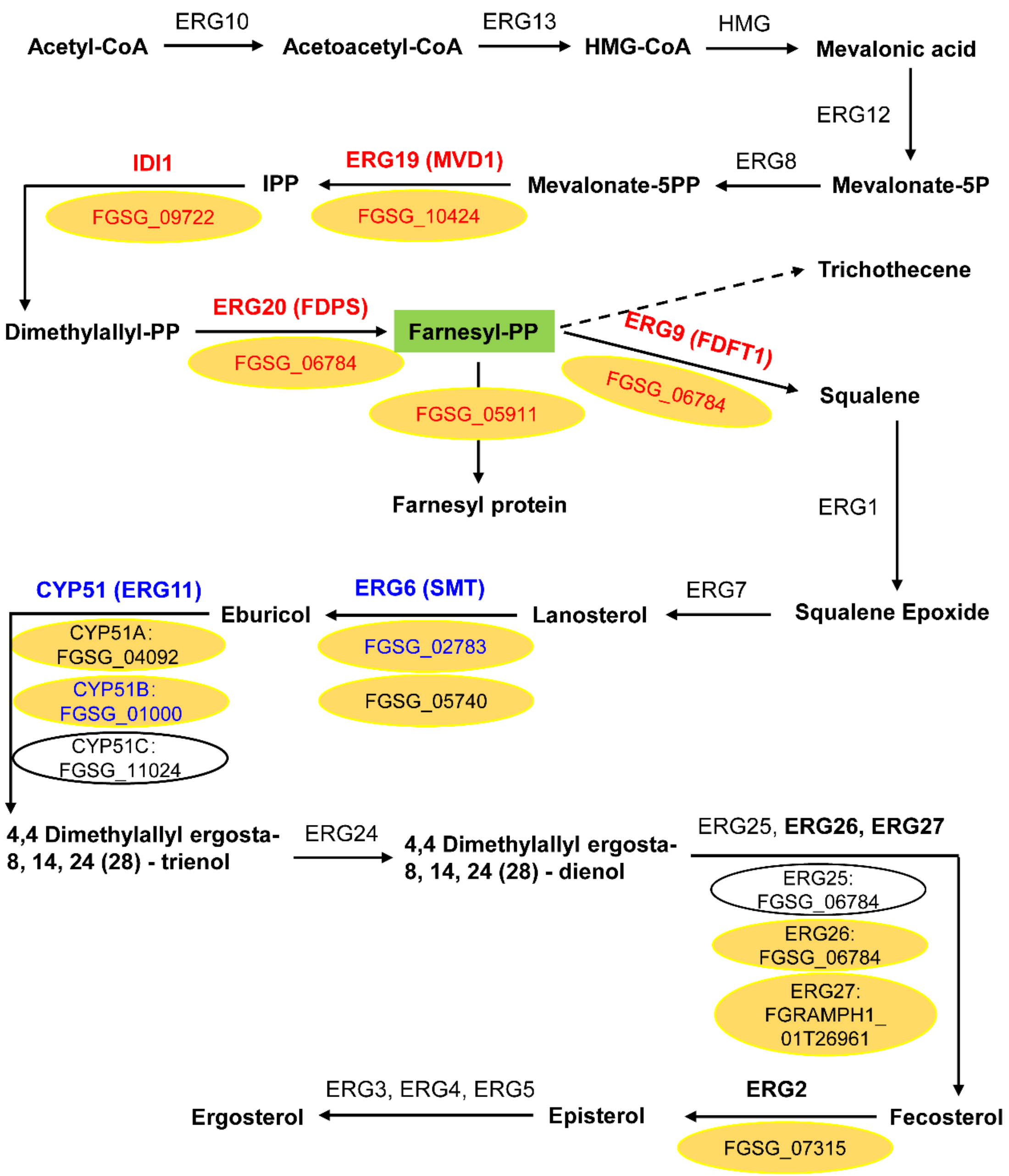

Figure S5. The sterol biosynthesis pathway based on the KEGG pathway fgr00900 and fgr00100, and the report of Fan et al [53]. All the enzymes with identified Khib sites are labeled in bold, and the corresponding gene locus is indicated on yellow background. Enzyme names with enhanced Khib sites in response to tebuconazole are in red, while those with suppressed Khib sites are in blue. Khib proteins not affected by tebuconazole treatment are indicated in black.

Raw file	Scan	Method	Score	m/z
F9111TP2o	19595	FTMS; HCD	65.92	841.43

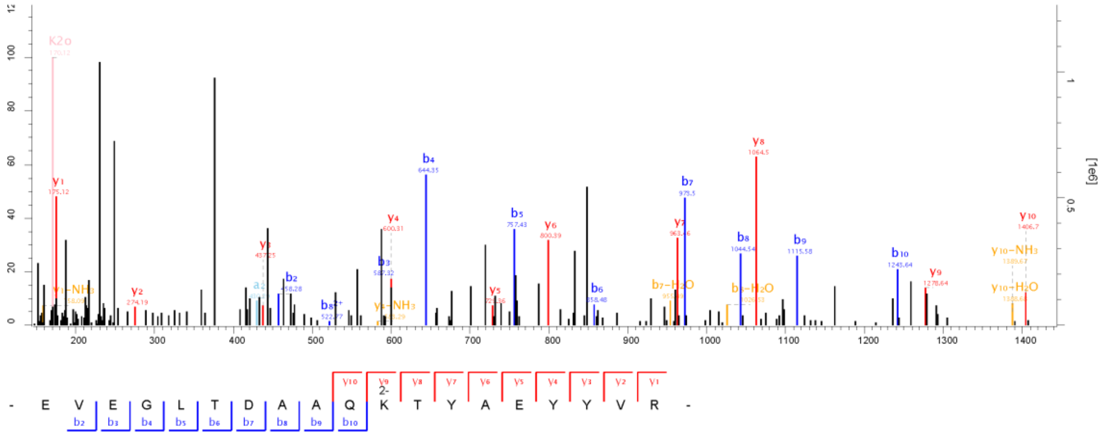

Raw file	Scan	Method	Score	m/z
F9111TP2o	23065	FTMS; HCD	149	912.49

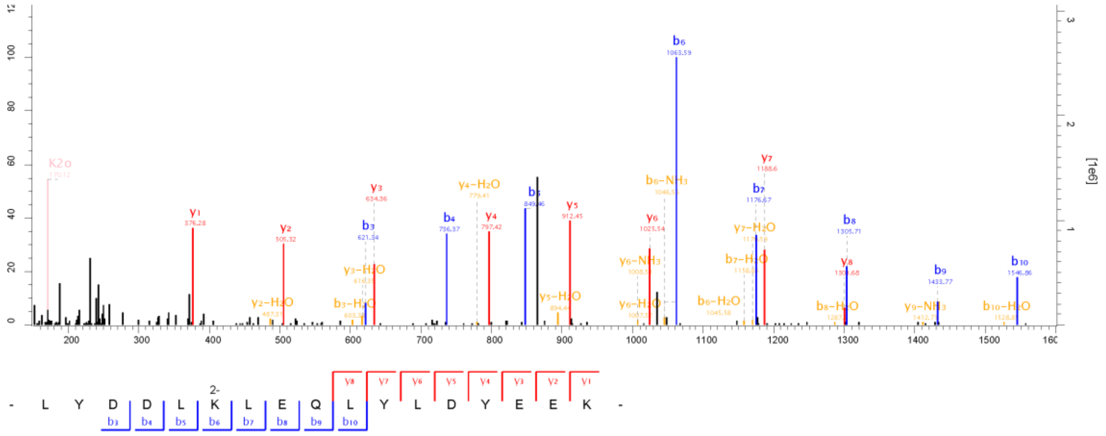

Raw file	Scan	Method	Score	m/z
F9111TP2o	9048	FTMS; HCD	99.54	570.31

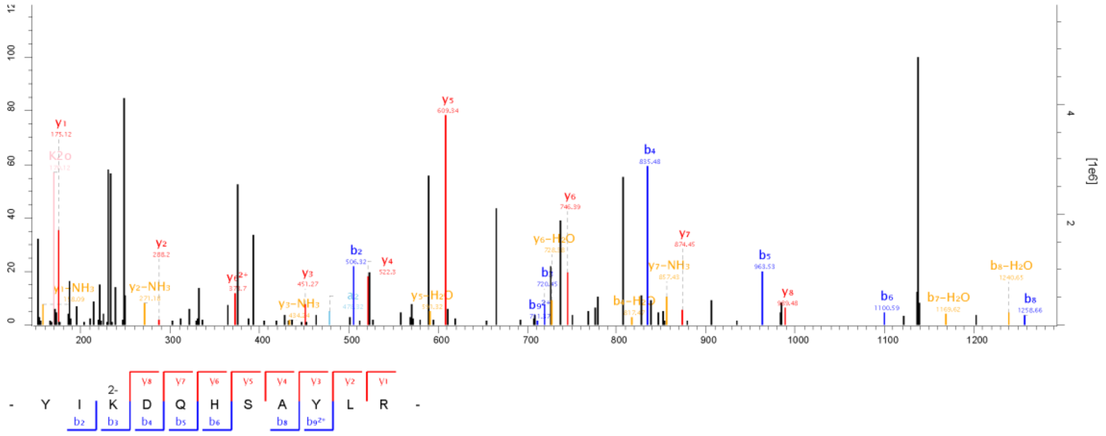

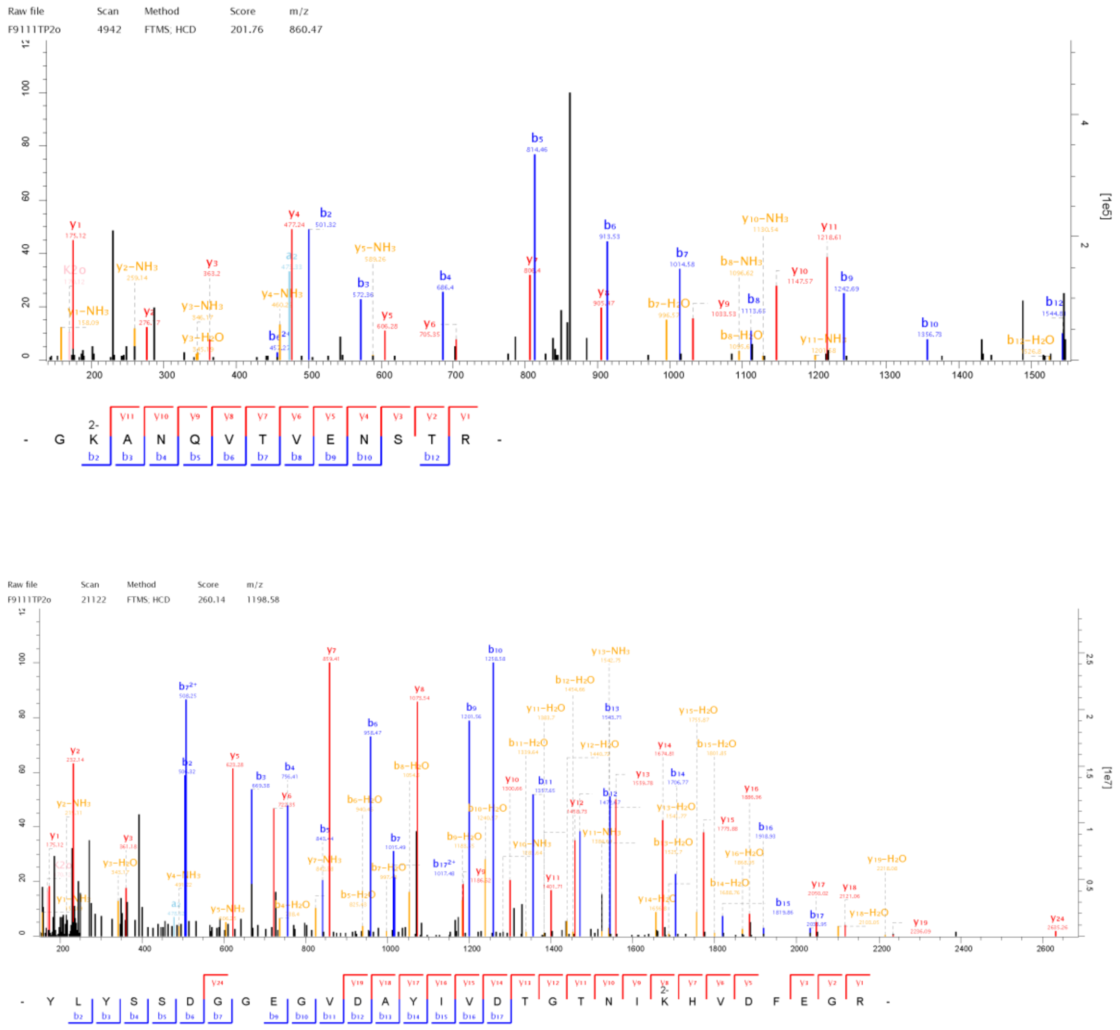

Figure S6. Several representative MS/MS spectra and peak assignments for the Khib peptides.
